# A systematic review and meta-analysis of social cognitive theory-based physical activity and/or nutrition behavior change interventions for cancer survivors

**DOI:** 10.1007/s11764-014-0413-z

**Published:** 2014-11-29

**Authors:** Fiona G. Stacey, Erica L. James, Kathy Chapman, Kerry S. Courneya, David R. Lubans

**Affiliations:** 1 School of Medicine and Public Health, University of Newcastle, W4, HMRI Building, Callaghan, NSW 2308 Australia; 2 Hunter Medical Research Institute, Callaghan, NSW Australia; 3 Priority Research Centre for Health Behavior, University of Newcastle, Callaghan, NSW Australia; 4 Priority Research Centre in Physical Activity and Nutrition, University of Newcastle, Callaghan, NSW Australia; 5 Cancer Council New South Wales, Woolloomooloo, NSW Australia; 6 Faculty of Physical Education and Recreation, University of Alberta, Edmonton, Alberta Canada; 7 School of Education, University of Newcastle, Callaghan, NSW Australia

**Keywords:** Cancer, Physical activity, Nutrition, Systematic review, Social cognitive theory

## Abstract

**Purpose:**

Little is known about how to improve and create sustainable lifestyle behaviors of cancer survivors. Interventions based on social cognitive theory (SCT) have shown promise. This review examined the effect of SCT-based physical activity and nutrition interventions that target cancer survivors and identified factors associated with their efficacy.

**Methods:**

A systematic search of seven databases identified randomized controlled trials that (i) targeted adult cancer survivors (any point from diagnosis); (ii) reported a primary outcome of physical activity, diet, or weight management; and (iii) included an SCT-based intervention targeting physical activity or diet. Qualitative synthesis and meta-analysis were conducted. Theoretical constructs and intervention characteristics were examined to identify factors associated with intervention efficacy.

**Results:**

Eighteen studies (reported in 33 publications) met review inclusion criteria. Meta-analysis (*n* = 12) revealed a significant intervention effect for physical activity (standardized mean difference (SMD) = 0.33; *P* < 0.01). Most studies (six out of eight) that targeted dietary change reported significant improvements in at least one aspect of diet quality. No SCT constructs were associated with intervention effects. There were no consistent trends relating to intervention delivery method or whether the intervention targeted single or multiple behaviors.

**Conclusions:**

SCT-based interventions demonstrate promise in improving physical activity and diet behavior in cancer survivors, using a range of intervention delivery modes. Further work is required to understand how and why these interventions offer promise for improving behavior.

**Implications for Cancer Survivors:**

SCT-based interventions targeting diet or physical activity are safe and result in meaningful changes to diet and physical activity behavior that can result in health improvements.

## Background

Due to population growth and aging and improved cancer detection and treatment, the number of cancer survivors is increasing [1, 2]. Many cancer survivors experience side effects from treatment and are at risk for secondary cancers and other chronic diseases such as cardiovascular disease and diabetes [3]. Several systematic reviews and meta-analyses have synthesized the findings of physical activity (PA) interventions in cancer survivors [4–9]. These reviews concluded that being physically active improved fitness, strength, body composition, fatigue, anxiety, depression, self-esteem, physical function, bone health, and quality of life [5, 6, 8, 9] and reduced risk of cancer recurrence and mortality among survivors of breast, bowel, prostate, and ovarian cancer [9, 10]. Dietary interventions report improved physical functioning [11, 12] and weight loss [13], lower levels of depression [12], and a reduction in new cancer events [13]. Diet and PA also play a role in energy balance and weight management, an independent predictor of cancer risk, and risk of recurrence and mortality [14] and contribute to the development of other chronic diseases, like diabetes and cardiovascular disease [15–17]. For these reasons, guidelines recommend PA (both aerobic and resistance), healthy diet, and healthy weight management for improving the health and well-being [8, 9, 14, 18–21] of cancer survivors across all phases of the cancer continuum [22]. While weight management is not a lifestyle behavior, it is the key target of lifestyle behavior strategies. Despite the potential impact of behavior on improved health outcomes, cancer survivors’ compliance with health recommendations remains less than optimal and is similar to the general population [23–25].

Effective diet and PA interventions have the potential to improve cancer survivors’ health, but little is known about what interventions work best. Interventions based on behavioral theory are reported to be more effective than atheoretical approaches [26, 27]. Theory-based research provides a framework for the development and evaluation of interventions [28] and facilitates understanding of the factors that mediate behavior change and the reasons why the intervention worked or failed [29, 30]. Social cognitive theory (SCT) is one promising theory for use in behavior change interventions [31], particularly as it provides a framework for understanding why people make and maintain health behaviors. The key constructs of SCT include the following: (1) knowledge of health risks and benefits; (2) perceived self-efficacy that a person can control their own health habits; (3) the expected costs and benefits or outcome expectations; (4) health goals, both proximal and distal intentions to engage in the behavior; (5) perceived facilitators and social support; and (6) barriers to making changes [31]. In 2004, Bandura reported a framework that specified the key determinants and the interplay between the key constructs (known as “reciprocal determinism”). Knowledge of health risks and benefits sets the scene for possible behavior change; however, it is not enough to prompt behavior change alone [31]. Self-efficacy influences outcome expectations and barriers/facilitators, and all constructs influence goals [31]. All constructs influence behavior and motivation and are influenced by the environment [31]. Self-efficacy is the central construct in SCT because it influences behavior directly, through belief in their ability to apply skills effectively in difficult situations, and indirectly, through influence on goals, outcome expectations, and barriers and facilitators [31, 32]. Self-efficacy is a major influence on motivation by determining the goals people set for themselves, the strength of commitment to them, and the outcomes they expect for their efforts [32]. Self-efficacy allows the individual to gain knowledge and develop skills, and as self-efficacy increases, people expect positive outcomes, overcome barriers, and show motivation and commitment to goals [32]. SCT constructs explain 40–71 % of the variance in PA behavior in adults [33–37] and have been shown to explain dietary behavior in adults, explaining 14–35, 22–53, and 36–61 % of the variance in fat, fiber, and fruit and vegetable intake [37, 38]. SCT also offers principles on how to motivate people to make positive behavior change [31]. Previous meta-analysis of health outcomes trials with cancer survivors concluded that SCT-based interventions resulted in improvements in global affect, depression, social outcomes, objective physical outcomes, and specific quality of life outcomes [39]. However, little is known about whether interventions based on SCT can positively impact on PA and diet behaviors, and what constructs and intervention characteristics are associated with increased behavior change.

There are currently no systematic reviews including multiple cancer types that synthesize both PA and diet behavior change interventions. While there is significant evidence supporting the impact of diet and PA behavior on health outcomes, there is a need to move to interventions that test how to motivate cancer survivors to make positive sustainable behavior change. Current evidence suggests that cancer survivors do not maintain PA behavior after participating in a supervised PA intervention [40, 41]. This review examined PA and diet behavior change interventions based on SCT in cancer survivors with mixed diagnoses both during and after completion of cancer treatment [42].

### Aim

This systematic review and meta-analysis aimed to (1) synthesize randomized controlled trials (RCTs) evaluating the efficacy of SCT-based behavior change interventions on PA and/or diet behaviors for cancer survivors of mixed diagnoses and (2) identify successful strategies for behavior change that can be used to guide intervention development. Of importance for a review with this aim is the definition of what constitutes a behavior change trial. We have used the reference defined by Courneya [43], that is, trials where the primary outcome is behavior (as opposed to a health outcomes trial where the primary outcome is quality of life, fatigue, etc.).

## Method

### Search strategy

The review was guided by the PRISMA statement [44]. Studies were identified by structured database search from inception until September 2014, in PsycINFO, CINAHL, Cochrane Central Register of Controlled Trials, Embase, Medline, SportDiscus, and Web of Science using the following search strings:

1. (Cancer survivor) or (cancer patient) or cancer.
2. Nutrition or diet or food or fruit or vegetable.
3. (Physical activity) or exercise or weight or aerobic or (strength training) or (resistance training) or walking.
4. (Social cognitive theory) or (social cognitive) or (social learning theory) or (behavio#r change theor*). Strings were made up of 1 + (2 or 3) + 4.

A sample search strategy is listed in the Appendix 1. Searches were limited to English language articles and those that targeted humans. Study titles were screened for eligibility by a single reviewer (FS). Full text of the remaining titles was obtained and screened in hierarchical order with studies excluded at the first reason for exclusion (FS).

1. Participants: adults aged 18 years or older, diagnosed with any cancer (at any point from diagnosis)
2. Outcomes: primary outcome of PA or diet or body weight (loss, or maintenance)
3. Intervention:
  - Any intervention designed to influence any type of PA or diet quality
  - Based on Bandura’s SCT [31], or explicitly described and referenced any SCT component (such as “self-efficacy”)
4. Comparator: any parallel control group
5. Study design: RCTs

### Data extraction

Data extraction was conducted by one author (FS), and the extracted data was independently checked by a second author (EJ, DL, or KC). Disagreements were resolved by consensus. Data extraction forms were developed, piloted with one trial, and amended (FS). The following data were extracted: study population and eligibility, behavior change outcomes and follow-up periods, intervention characteristics, and how the theory constructs were operationalized and assessed. Where authors indicated a trial protocol number, the protocol was retrieved, but no other attempt was made to obtain unpublished trial information.

### Synthesis of results

Separate meta-analyses were planned for PA and dietary outcomes. However, due to heterogeneity in dietary outcomes, meta-analyses were conducted only for studies that reported the effect of the intervention on total PA using RevMan version 5.1 [45]. As recommended by the Cochrane Collaboration, posttest means and their standard deviations were used in the analysis. Intention-to-treat data was extracted from papers. When studies compared multiple treatment groups with a single control group (*n* = 2), the sample size of the control group was divided to avoid double counting. All data were considered continuous, but as PA was measured using various methods, we report the standardized mean difference (SMD) and their 95 % confidence intervals. Statistical heterogeneity was examined using chi-squared and the *I* ^2^ index tests. A guide to the interpretation of heterogeneity based on the *I* ^2^ index is as follows: 0–40 % might not be important; 30–60 % may represent moderate heterogeneity; 50–90 % may represent substantial heterogeneity; and 75–100 % considerable heterogeneity [46].

Subgroup analyses comparing the number of behaviors targeted (multiple behaviors compared to one only), cancer type, and number of theoretical constructs operationalized (self-efficacy compared to multiple theoretical constructs) were planned. However, the limited number of studies and heterogeneity of included trials did not allow for subgroup analyses.

### Risk of bias

Risk of bias was assessed using the McMaster Quality Assessment Tool [47], with a score of “strong,” “moderate,” or “weak” methodological quality assigned to each of six sections (1. selection bias; 2. study design; 3. confounders; 4. blinding; 5. data collection methods; 6. withdrawals and dropouts). A global rating was made based on the ratings from each of the six sections. As recommended, papers with no “weak” ratings were “strong” methodological quality; those with one “weak” rating were “moderate”; and those with two or more “weak” ratings were “weak” [47]. Risk of bias was undertaken by two independent reviewers (FS and EJ, or DL, or KC), with disagreements resolved by consensus (FS and EJ).

## Results

### Study selection

**Fig. 1:**
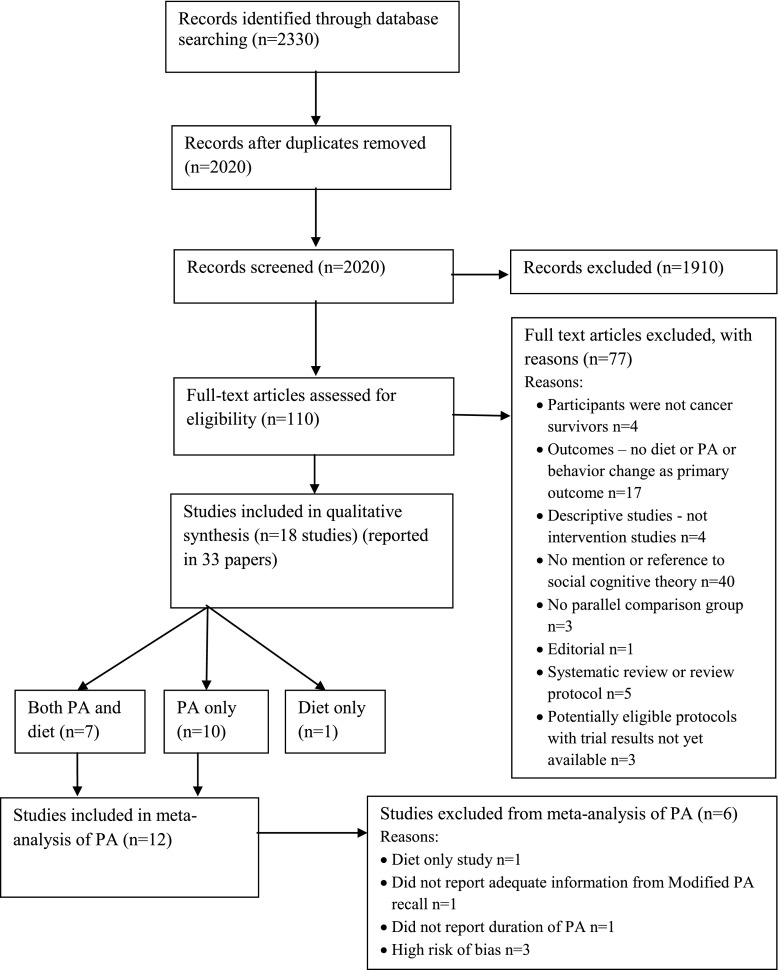
PRISMA flow diagram

Figure 1 shows the flow of studies through the review process and the reasons for exclusion. Database searches resulted in 2020 potentially relevant titles. The full text of 110 articles was assessed for eligibility, and 18 studies (reported in 33 publications) met inclusion for the review (Table 1). Studies were grouped and reported by intervention topic: PA only (ten trials), diet only (one trial), or multiple health behavior (PA and diet) (seven trials).

**Table 1:** Description of included trials

Study	Participants; mean age; cancer type; time since diagnosis	Intervention (type, intensity, duration)	Study design and evaluation	Outcomes	Results	Retention
PA-only studies						
Short et al. [54, 55] *Country*: Australia	*Participants*: *N* = 330 *Mean age*: 55 years *Cancer type*: breast *Time since diagnosis*: 41 months since active treatment (SD = 39)	*G1*: Standard recommendation control group received national PA guidelines brochure *G2*: tailored-print: computer-tailored A4 4-page newsletters (3) *G3*: targeted-print intervention: 54-page A5 booklet “Exercise for Health” (1) *Type*: aerobic PA, at least moderate intensity, for 30 min or more on most days of the week. In G2 and G3, participants were also encouraged to perform resistance training exercises 1–3 times per week *Intensity*: G2 had 3 newsletters over 12 weeks. G3 had 1 mailout over 12 weeks *Duration*: G2 received one newsletter each 6 weeks	*Study design*: 3 arm RCT *Follow-up*: 4, 10 months *Comparison group*: usual care	*Outcome measures*: Godin leisure time PA^a^, adherence to meeting aerobic and resistance training guidelines, mean daily steps (pedometer)	*Primary*: G2 reported statistically significant effect on self-reported resistance activity (*P* < 0.01) and on meeting the resistance training guidelines (*P* < 0.01). G2 and G3 reported nonsignificant improvements to self-reported aerobic activity. There was no significant effect for meeting the aerobic PA guidelines *Other behaviors*: nonsignificant increase in step counts for G2 and G3 participants. G1 step counts decreased	90 % (*n* = 299)
Valle et al. [56] *Country*: USA	*Participants*: *N* = 86 *Mean age*: 31.7 years *Cancer type*: 31 % hematologic; 20 % breast; 15 % gynecologic *Time since diagnosis*: 58.2 months (SD = 44.0)	*G1*: Facebook-based self-help comparison group *G2*: Facebook-based intervention group with weekly behavioral lesson on PA and behavioral strategies (12) (FITNET) *Type*: 150 min of moderate intensity PA per week *Intensity*: 12 weekly behavioral lessons (sent via Facebook message), discussion questions posted on Facebook (16 total), resources and reminders each posted once per week (24) *Duration*: minimum of 4 contacts per week over 12 weeks	*Study design*: 2 arm RCT *Follow-up*: 12 weeks *Comparison group*: self-directed Facebook group	*Outcome measures*: Godin leisure time exercise questionnaire^a^, intervention adherence and acceptability	*Primary*: significant difference between groups in estimated change in light PA mins per week over 12 weeks. G2 participants reported increases in mins of moderate to vigorous PA and total PA; however, these were not significant	77 % (*n* = 66)
Rogers et al. [51, 68–70] *Country*: not explicitly stated. Authors based in Illinois, USA	*Participants*: *N* = 41 *Mean age*: 53 years *Cancer type*: breast *Time since diagnosis*: 34 months since surgery (0.7–134)	*G1*: wait-list control *G2*: discussion group sessions (6), individual-supervised exercise (12), individual face-to-face counseling (3), transition to home-based program *Type*: moderate intensity with the aim of building up to 150 min of moderate walking per week *Intensity*: 21 sessions over 3 months *Duration*: multiple exposure (minimum weekly)	*Study design*: 2 arm RCT *Follow-up*: 3, 6 months *Comparison group*: wait-list control	*Outcome measures*: total activity counts^a^, steps, mins of moderate-vigorous PA (accelerometer); Godin leisure time PA	*Primary*: significant increase in total activity counts (mean difference = 72,103). Effect size *d* = 1.02 (*P* = 0.004) as measured by accelerometer at 3 months and remained significant at 6 months (mean difference = 61,651; *P* = 0.06) *Other behaviors*: significant increase in moderate and vigorous minutes (*d* = 0.57; *d* = 0.54 (*P* = 0.09)). Nonsignificant increase in self-reported moderate and vigorous activity (*d* = 0.16; *P* = 0.63)	92 % (*n* = 38)
Pinto et al. [50, 71]; Rabin et al. [72] *Country*: not explicitly stated. Authors based in Rhode Island, USA	*Participants*: *N* = 86 *Mean age*: 53 years *Cancer type*: early stage breast cancer *Time since diagnosis*: 1.74 years (SD 1.49)	*G1*: contact control, weekly phone call and cancer survivorship written sheets *G2*: weekly telephone counseling (12) and customized written feedback (4), home exercise logs, pedometer *Type*: moderate intensity PA (55–65 % of maximum heart rate) walking gradually building up to goal of 30 min of accumulated PA per day on at least 5 days per week *Intensity*: 16 contacts over 3 months *Duration*: multiple exposure (minimum weekly)	*Study design*: 2 arm RCT *Follow-up*: 12 weeks, 6 months, 9 months *Comparison group*: attention control	*Outcome measures*: 7 day PA recall^a^, 1 mile walk test^a^, accelerometer^a^, percentage of body fat (skinfold thickness)^a^, body mass index^a^	*Primary*: significant increase in mins of PA, and 1 mile walk test (*P* < 0.001). Significant between group differences were evident for total mins of PA on 7 day PA recall (*P* < 0.001), higher weekly mins of moderate intensity PA (*P* < 0.001), higher total energy expenditure (*P* < 0.001) at 12 weeks No difference in body mass index or percentage of body fat *Other behaviors*	95 % (*n* = 82)
Bennett et al. [57] *Country*: not explicitly stated. First author based in New Zealand; co-authors based in Portland, USA	*Participants*: *N* = 56 *Mean age*: intervention 56 years; control 60 years *Cancer type*: any *Time since diagnosis*: 4.8 years (SD 3.0) intervention	*G1*: contact control *G2* **:** single face-to-face counseling (30 min) with 3 follow-up telephone calls (20 min per call), pedometer *Type*: physical activity aim to reach 30 min of moderate intensity PA on most days of week *Intensity*: 4 contacts over 18 weeks *Duration*: multiple exposure (at least 2 weeks apart)	*Study design*: 2 arm RCT *Follow-up*: 3, 6 months *Comparison group*: attention control	*Outcome measures*: Community Healthy Activities Model Program for Seniors (CHAMPS) (caloric expenditure per week in kilocalories per week)^a^	*Primary*: significant increase in PA at 6 months (*d* = 0.55; *P* < 0.05) with a difference in PA increase over time of 1159 kcal per week between the two groups *Other behaviors*	Intervention. 71.4 % (*n* = 20); control, 92.9 % (*n* = 26)
Matthews et al. 2007 [58] *Country*: USA	*Participants*: *N* = 36 *Mean age*: intervention 51 years; control 57 years *Cancer type*: postmenopausal breast *Time since diagnosis*: 0.9 years (range 0.7–1) intervention	*G1*: wait-list control *G2*: face-to-face behavioral counseling (1) and telephone counseling (5) *Type*: walking (moderate intensity) building from 20 to 30 min per session, 3 times per week to 30–40 min per session, 5 times per week *Intensity*: 6 sessions over 12 weeks *Duration*: decreasing frequency over 12 weeks. Face-to-face counseling (30 min). Telephone counseling (10–15 min per call)	*Study design*: 2 arm RCT *Follow-up*: 6, 12 weeks *Comparison group*: wait-list control	*Outcome measures*: Community Healthy Activities Model Program for Seniors (CHAMPS) (energy expenditure MET-h per week)^a^, accelerometer (subsample only), 21-item diet habits questionnaire, 19-item fruit and vegetable screener	*Primary*: significant increase in self-reported walking (*P* = 0.01), MET-h per week (*P* = 0.01) with difference of 10.2 MET-h per week of walking at 12 weeks between the groups *Other behaviors*: No significant changes to fruit and vegetable consumption and overall dietary habits and no significant changes were noted, although the data was not shown	Not reported
Ligibel et al. [49] *Country*: USA	*Participants*: *N* = 121 *Mean age*: 54 years *Cancer type*: breast, colon or rectal cancer *Time since diagnosis*: not reported	*G1*: usual care, offered one consultation with exercise trainer *G2*: semistructured telephone counseling and participant workbook *Type*: physical activity *Intensity*: 10**–**11 semistructured telephone calls (30–45 min each) over 16 weeks *Duration*: decreasing frequency over 16 weeks	*Study design:* 2 arm RCT *Follow-up*: 16 weeks *Comparison group*: usual care	*Outcome measures*: 7 day PA recall interview (change in minutes of weekly PA)^a^	*Primary*: nonsignificant increase in physical activity minutes per week by 40 min (*P* = 0.13), and MET-h per week by 2 h (*P* = 0.23) *Other behaviors*	Intervention, 79 % (*n* = 48); control, 85 % (*n* = 51)
Wang et al. [52] *Country*: Taiwan	*Participants*: *N* = 72 *Mean age*: 50 years *Cancer type*: newly diagnosed breast, scheduled to start chemotherapy *Time since diagnosis*: First meeting is 24 h prior to participant surgery	*G1*: usual care *G2*: weekly telephone call and weekly individual face-to-face meetings (heart rate monitor, pedometer, exercise diary, and role model story) *Type*: home-based walking program of low to moderate intensity from 40 to 60 %, 3 to 5 times per week, at least 30 min per session or the accumulation of 30 min per session *Intensity*: 12 telephone and face-to-face contacts over 6 weeks *Duration*: 2 sessions per week	*Study design*: 2 arm RCT *Follow-up*: 24 h prior to day 1 of chemotherapy, the day of chemotherapy mid-cycle, 6 weeks *Comparison group*: usual care	*Outcome measures*: exercise behavior (Godin leisure time questionnaire)^a^	*Primary*: significant increases in physical activity at all follow-up time points for the intervention group (all *P* < 0.05). The difference between the groups was 62.7, 58.2, and 38.8 min at the second, third, and fourth follow-up (*P* < 0.001) *Other behaviors*	Intervention, 86 % (*n* = 30); control, 86 % (*n* = 32)
Pinto et al. [53] *Country*: USA	*Participants*: *N* = 46 *Mean age*: 57 years *Cancer type*: colon or rectal cancer *Time since diagnosis*: 3.1 years (SD 1.6) (intervention)	*G1*: contact control group offered written material at study completion *G2*: one face-to-face appointment, one weekly telephone call (12), weekly PA and cancer survivorship tip sheet (12), feedback letter summarizing progress (4), pedometer *Type*: moderate intensity home-based PA with goal to perform moderate intensity activity aerobic activities at 64–76 % of estimated maximum heart rate *Intensity*: 29 contacts over 12 weeks *Duration*: 2 contacts per week minimum	*Study design*: 2 arm RCT *Follow-up*: 3, 6, 12 months *Comparison group*: contact control, offered written materials at study completion	*Outcome measures*: 7 day PA recall (self-reported PA)^a^, Community Healthy Activities Model Program for Seniors (CHAMPS), accelerometer	*Primary*: Significant increase in PA at 3 months in intervention (*d* = 1.93) (*P* = 0.02), but increases were not maintained at 6 and 12 month follow-ups. There was a significant difference between groups at 3 months by 117 min/week (*P* < 0.05) but not at 6 or 12 months *Other behaviors*	Intervention, 95 % (*n* = 19); control, 88 % (*n* = 23)
Hatchett et al. [48] *Country*: not explicitly reported. Lead author: Mississippi, USA	*Participants*: *N* = 85 *Mean age*: not reported *Cancer type*: breast cancer *Time since diagnosis*: 44 % between 0 and 20 months; 27 % between 21 and 40 months; 19 % between 41 and 70 months	*G1*: wait-list control *G2*: email messages (8), access to an e-counselor (experienced exercise physiologist) *Type*: physical activity (emails focused on changing SCT constructs) *Intensity*: total 8 email messages, weekly for 5 weeks, then fortnightly for next 6 weeks *Duration*: once per week (5 weeks), then fortnightly for 6 weeks for a total of 8 messages over 12 weeks	*Study design*: 2 arm RCT *Follow-up*: 6, 12 weeks *Comparison group*: wait-list control	*Outcome measures*: 7 day PA recall^a^	*Primary*: At 12 weeks, for total days of exercise, there was a significant difference between the groups (*P* < 0.001) with the intervention reporting 2.05 more days of exercise compared to the control group (*P* < 0.001) *Other behaviors*	Intervention, 88.4 % (*n* = 38); control, 85.7 % (*n* = 36)
Diet only						
Parsons et al. [59] *Country*: USA	*Participants*: *N* = 43 *Mean age*: 64 years *Cancer type*: prostate *Time since diagnosis*: not reported—receiving only active surveillance as treatment	*G1*: standard care control *G2*: telephone counseling *Type*: diet (7 servings/day vegetables; 2 servings/day whole grains, 1 serving/day beans/legumes) *Intensity*: total of 13 sessions *Duration*: 13 structured telephone counseling sessions over 6 months. Call duration of 25–50 min	*Study design*: 2 arm RCT *Follow-up*: 6 months *Comparison group*: usual care	*Outcome measures*: 24 h dietary recall^a^, blood samples (plasma carotenoid concentration)^a^	*Primary*: Total vegetable and tomato product intake significantly increased in the intervention (*P* < 0.05). No significant changes in fruit, whole grain, beans, grams of fiber per day, or fat intake *Other behaviors*	96.7 % (*n* = 42)
Multiple behavior studies						
Demark-Wahnefried et al. STRENGTH trial [60] *Country*: USA	*Participants*: *n* = 90 *Mean age:* 41.8 years *Cancer type*: premenopausal breast *Time since diagnosis*: not reported, intervention occurs during chemotherapy	*G1*: attention control (calcium-rich diet) *G2*: calcium-rich diet and exercise (telephone counseling contacts (14), exercise equipment, heart rate monitor, workbook, videotape) *G3*: calcium-rich diet and exercise and high fruit and vegetable, low-fat diet (resources provided to G2 + encouraged to maintain high fruit and vegetable, low-fat diet to reduce energy density of the diet). Goal levels of <20 % of energy from fat, and >5 servings fruit and vegetables per day *Type*: diet (high fruit and vegetable, low fat) and exercise (aerobic and strength training) (aerobic exercise >3 times/week, strength training every other day) *Intensity*: multiple contacts (at least fortnightly) *Duration*: 14 telephone counseling contacts (10–30 min) over 6 months	*Study design*: 3 arm RCT *Follow-up*: 3, 6 months *Comparison group*: attention control	*Outcome measures*: % body fat (whole body DXA scans)^a^, 144-item diet history questionnaire, Longitudinal Study Physical Activity Questionnaire (MET-h/week), accelerometer (kcal/day)	*Primary*: Consistent increases for all measures of adiposity were observed over time and among all groups. G3 had significantly lower scores for % of body fat (minus the trunk) (*P* < 0.05) *Other behaviors*: no significant changes in physical activity over time or between study arms There were no differences in energy intake among study arms. However, G3 exhibited higher fruit and vegetable intakes (by 1.7 serves) and lower fat intakes (reduction of 5.2 % calories from fat) at 6 months	91.2 % (*n* = 82)
Campbell et al. [61]; Reedy et al. [111]; Ko et al. [112] *Country*: USA	*Participants*: *N* = 922 (*n* = 266 colorectal cancer survivors) *Mean age*: 66.5 years *Cancer type*: colorectal cancer *Time since diagnosis*: 7.6 %: less than 1 year ago; 29 %: 1–2 years ago; 57 %: 2–5 years ago	*G1*: generic health education (2 mailings) and tailored-print newsletters (4) after follow-up completed *G2*: 4 tailored-print newsletters *G3*: 4 telephone calls (20 min duration) *G4*: 4 tailored-print newsletters +4 telephone calls (20 min duration) *Type*: diet, physical activity, colorectal cancer screening *Intensity*: multiple exposure (less than monthly) *Duration*: 1 year	*Study design*: 4 arm RCT. 2 × 2 design—stratified by colorectal cancer and noncolorectal cancer status *Follow-up*: 6, 12 months *Comparison group*: usual care with tailored newsletters at study completion	*Outcome measures*: modified Block Food Frequency Questionnaire (36 item)^a^, 2-item fruit and vegetable screening questions^a^, modified 7 day PA recall (moderate to vigorous PA score)	*Primary*: There were no significant changes in fruit and vegetable consumption in colorectal cancer survivors, using the Food Frequency Questionnaire. There was a nonsignificant increase in G2 intervention by a mean of 1.0 serves/day Using the 2-item screening questions, all 3 intervention groups showed statistically significant increases among colorectal cancer survivors *Other behaviors*: No significant change on physical activity, and participants in all 4 groups were less active at follow-up compared to baseline	79.7 % (*n* = 735) from total sample
Von Gruenigen et al. [65, 73] *Country*: USA	*Participants*: *N* = 45 *Mean age*: 55 years *Cancer type*: endometrial cancer *Time since diagnosis*: 20.6 months median (intervention)	*G1*: standard care *G2*: face-to-face group sessions, telephone, or written newsletters *Type*: weight loss, PA, eating behaviors *Intensity*: weekly contact *Duration*: total of 21 sessions of face-to-face (11), telephone (5), written newsletters (5) over 6 months	*Study design*: 2 arm RCT *Follow-up*: 3, 6, 12 months *Comparison group*: usual care	*Outcome measures*: weight change (kilograms)—measured^a^, PA using Leisure Score Index of the Godin leisure time exercise questionnaire (frequency per week on Leisure Score Index for mild, moderate, strenuous PA), 3 day food record (vitamin C and folate as marker of fruit and vegetable intake, kilocalories)	*Primary*: The mean difference in weight change between the two groups was −4.9 kg (*P* = 0.018) at 12 months. The control group did not demonstrate any significant changes in weight from baseline. Mean weight change expressed as a percentage from baseline to 12 months was −3.1 % in the intervention compared to 1.0 % in the control group (mean difference −4.1 %, *P* = 0.020) *Other behaviors*: At 12 months, there was a significant difference in Leisure Score Index between groups (mean group difference 17.8, *P* = 0.002) There were no significant changes in diet. The intervention group had a lower energy intake (kilocalories) but was not statistically significant from the control group	Intervention, 78 % (*n* = 18); control, 90 % (*n* = 20)
Von Gruenigen et al. [67] *Country*: USA	*Participants*: *N* = 75 *Mean age*: 58.0 years *Cancer type*: endometrial cancer *Time since diagnosis*: 20.7 months	*G1*: standard care *G2*: face-to-face group sessions, individual physician counseling, newsletters, telephone, and email contact with registered dietician. Received pedometer, heart rate monitor, hand and ankle weights *Type*: weight loss, PA, resistance exercises, diet quality *Intensity*: weekly (10), then biweekly (6) group sessions. Physician counseling at 3, 6, and 12 months *Duration*: minimum of 19 contacts for 12 months	*Study design*: 2 arm RCT *Follow-up*: 3, 6, 12 months *Comparison group*: standard care received one information brochure	*Outcome measures*: measured weight^a^ and height, waist circumference, hip circumference, Godin leisure time exercise questionnaire, 2 × 24 h dietary recalls, pedometer step counts	*Primary*: significant differences for weight change from baseline to 3, 6, and 12 months (*P* < 0.001). Mean (95 % CI) difference between groups at 6 months was −4.4 kg [−5.3, −3.5], *P* < 0.001 and at 12 months was −4.6 kg [−5.8, −3.5], *P* < 0.001. Mean percent weight change in the intervention was −4.1 % as compared to −0.8 % in controls at 6 months and −3.0 % and +1.4 % at 12 months *Other behaviors*: mean (95 % CI) difference in change at 6 months was 100 min per week [6, 94], *P* = 0.038 and at 12 months was 89 min per week [14, 163], *P* = 0.020. Mean change in pedometer step counts from baseline to 6 months was 2353 in the intervention group versus −9.4 steps per day in the usual care group (difference of [95 % CI] of 2362 (494, 4230); *P* = 0.015) Mean difference in change in total fruit and vegetable intake was 0.91 servings per day (*P* < 0.001) at 6 months and 0.92 (*P* < 0.001) at 12 months. Mean difference in change in kilocalories between groups was −228.8, −217.8, and −187.2 kcal at 3, 6, and 12 months (*P* < 0.001)	78.7 % (*n* = 59). Intervention, 85.4 % (*n* = 35); control, 70.6 % (*n* = 24)
Demark-Wahnefried et al. [62]; Demark-Wahnefried et al. [63]; Mosher et al. [78]; Wilkinson et al. [113]; Christy et al. [74].—FRESH START *Country*: USA	*Participants*: *N* = 543 *Mean age*: 57 years *Cancer type*: breast, prostate *Time since diagnosis*: 3.83 months (SD 2.74)	*G1*: attention control *G2*: tailored-print newsletters and workbook *Type*: diet and physical activity *Intensity*: initial workbook and (6) tailored newsletters every 7–9 weeks for 10 months *Duration*: total of 7 contacts for 10 months	*Study design*: 2 arm RCT *Follow-up*: 1 year, 2 years *Comparison group*: attention control	*Outcome measures*: number of goal behaviors practiced (percentage adopting goal behavior in at least 2 areas)^a^, 7 day PA recall, diet history questionnaire, Diet Quality Index mean score	*Primary*: both arms significantly improved their lifestyle behaviors (*P* < 0.05). Significant difference between groups in practice of 2 or more goal behaviors (*P* < 0.0001) (16 % greater in intervention participants) *Other behaviors*: significant differences between groups in exercise minutes per week (*P* = 0.02) (+20 min/week intervention), fruit and vegetables per day (*P* = 0.01) (+0.5 servings intervention), total fat (*P* < 0.0001) (−2.3 % intervention), saturated fat (*P* < 0.0001) (−1.0 %)	Intervention, 93.4 % (*n* = 253); control, 97.8 % (*n* = 266)
Djuric et al. [64] *Country*: USA	*Participants*: *N* = 40 *Mean age*: 52 years *Cancer type*: breast *Time since diagnosis*: not reported although either scheduled for or starting chemotherapy in the next 2 weeks	*G1*: control group received written diet and physical activity materials and pedometer (same as G2), and bimonthly study newsletters *G2*: written diet and physical activity materials, pedometer, telephone counseling (by a dietician trained in motivational interviewing) *Type*: high fruit and vegetable, low-fat diet, weight control, 30 min per day of moderate-to-vigorous PA *Intensity*: multiple contacts (at least monthly) *Duration*: total of 19 calls, written materials, and pedometer, over 12 months	*Study design*: 2 arm RCT *Follow-up*: 6, 12 months *Comparison group*: attention control with written materials and pedometer (same as the intervention group)	*Outcome measures*: measured weight and body fat^a^, 19-item fruit and vegetable screener, 17-item percentage of energy from fat, 24 h diet recall, Women’s Health Initiative validated PA questionnaire	*Primary*: the percentage of body fat increased by 1.2 % in the control group and decreased by 0.07 % in the intervention group. Weight decreased by 0.8 kg at 12 months *Other behaviors*: Total physical activity increased to a mean of 364 min per week and moderate/vigorous activity increased to a mean of 315 min per week at 12 months, slightly below the target of 350 min per week of moderate/vigorous activity. For fruit and vegetable intakes from unannounced recalls, the number of servings/day increased only in the telephone arm, and the mean reported intake at 12 months was just above the minimum intervention goal of 7 servings per day, not counting potatoes. There was a significant increase in fruit and vegetable servings by 3.1 serves from baseline to 12 months	Intervention. 65 % (*n* = 13); control. 85 % (*n* = 17)
Djuric et al. [66] *Country*: USA	*Participants*: *N* = 48 *Mean age*: mean: 51.7 years Cancer type: breast *Time since diagnosis*: not reported—although needed to have been diagnosed within the past 4 years	*G1*: standard care *G2*: weight watchers (free coupons to attend each week) (52) *G3*: telephone counseling by dietician (24 calls), and mailed written material (12) G4: weight watchers free coupons (52), dietician-delivered telephone counseling (24), mailed written material (12) *Type*: weight loss goal (10 % baseline weight over 6 months) by decreasing energy and fat intake, and 30–45 min moderate activity most days of the week *Intensity*: multiple contacts (minimum monthly) *Duration*: Total contacts varied from 36 (G3), 52 (G2), 88 (G4), in 12 month intervention	*Study design*: 4 arm RCT *Follow-up*: 3, 6, 12 months *Comparison group*: usual care	*Outcome measures*: weight^a^, 3 day food record, physical activity logs (self-reported intentional exercise)	*Primary*: significant difference in weight loss at 12 months for participants in G3 (mean 8 kg loss) and G4 (mean 9.4 kg loss). There was a nonsignificant loss of 2.5 kg in G2, and an increase of 0.85 kg in G1 (control group) *Other behaviors*: nonsignificant decreases in energy intake (kilocalories per day) in each of the three intervention groups (by 447–616 kcal per day), and nonsignificant decrease in fat intake (% of energy from fat) in the 3 intervention groups (by 2–11 %) at 12 months. The control group energy intake remained the same (decrease of 126 kcal per day), and increased fat intake (by 7 %) There was no difference in weight loss between women who self-reported intentional exercise (beyond daily activities), and those who reported no intentional activities in each study group	81.3 % (*n* = 39)

*G g*roup

^a^Denotes primary outcome

### Risk of bias assessment

There was initially 75 % agreement between authors on the study assessment criteria and full consensus was achieved after discussion. Risk of bias results are reported in Table 2. Of the ten PA-only studies, five were classified as strong methodological quality [48–52], three as moderate [53–56], and two as weak [57, 58]. The diet-only trial was classified as moderate [59]. In the seven multiple behavior studies, one was classified as strong [60], four were moderate [61–65], and two weak [66, 67]. Three trials were excluded from the meta-analysis [57, 58, 67] due to being weak.

**Table 2:** Risk of bias (assessed using McMaster Quality Assessment Tool) [47]

Study	(a) Selection bias	(b) Study design	(c) Confounders	(d) Blinding	(e) Data collection method	(f) Withdrawals and dropouts	Global rating
PA-only studies							
Short et al. [54, 55]	Weak	Strong	Strong	Moderate	Strong	Strong	Moderate
Valle et al. [56]	Weak	Strong	Strong	Moderate	Strong	Moderate	Moderate
Rogers et al. [51, 68]	Moderate	Strong	Moderate	Moderate	Strong	Strong	Strong
Pinto et al. [50, 71, 72]	Moderate	Strong	Strong	Moderate	Strong	Strong	Strong
Bennett et al. [57]	Weak	Strong	Strong	Weak	Strong	Moderate	Weak
Matthews et al. [58]	Weak	Strong	Strong	Weak	Moderate	Weak	Weak
Ligibel et al. [49]	Moderate	Strong	Strong	Moderate	Strong	Moderate	Strong
Wang et al. [52]	Moderate	Strong	Strong	Moderate	Strong	Strong	Strong
Pinto et al. [53]	Weak	Strong	Strong	Moderate	Strong	Strong	Moderate
Hatchett et al. [48]	Moderate	Strong	Strong	Moderate	Strong	Strong	Strong
Diet only							
Parsons et al. [59]	Moderate	Strong	Weak	Moderate	Strong	Strong	Moderate
Multiple behavior studies							
Demark-Wahnefried et al.—STRENGTH [60]	Strong	Strong	Strong	Moderate	Strong	Strong	Strong
Campbell et al. [61]	Weak	Strong	Strong	Moderate	Strong	Strong	Moderate
Von Gruenigen et al. [65, 73]	Weak	Strong	Strong	Moderate	Strong	Moderate	Moderate
Von Gruenigen et al. [67]	Weak	Strong	Strong	Weak	Strong	Moderate	Weak
Demark-Wahnefried et al.—FRESH START [62, 63, 78]	Weak	Strong	Strong	Moderate	Strong	Strong	Moderate
Djuric et al. [64]	Weak	Strong	Strong	Moderate	Strong	Moderate	Moderate
Djuric et al. [66]	Weak	Strong	Weak	Moderate	Strong	Strong	Weak

The most common areas with a high risk of bias were selection bias, confounders, and blinding. Eleven studies [53, 54, 56–58, 61–67] were rated as weak in selection bias category with less than 60 % of potentially eligible participants recruited. Two studies [59, 66] were rated as weak as the control of confounders was not described. No studies received a strong rating for blinding as (understandably given they are behavior change trials), all participants were aware of the research question, and if the outcome assessor was also aware of the intervention status of participants, studies were rated as weak methodological quality [57, 58, 67].

### Physical activity trials

There were ten trials that targeted PA alone [48–54, 56–58].

#### Participants

Ten PA trials reported a total of 960 participants (range 36–330). Six trials targeted breast cancer survivors [48, 50–52, 54, 58, 68–72], one targeted colorectal cancer survivors [53], one targeted both breast and bowel cancer survivors [49], and two included cancers of mixed diagnoses [56, 57]. Mean time since diagnosis was 3.1 years (range 0.9–4.9 years) [50, 51, 53, 54, 56–58]. Time since diagnosis was not reported in two studies [49, 52]; however, one trial reported participants were scheduled to begin chemotherapy [52]. All other trial participants had completed active cancer treatment (excluding hormone treatment). In five studies, only cancer survivors who were inactive or insufficiently active were eligible to participate [48–50, 57, 58]. Three trials used a wait-list control group design [48, 51, 58], three had attention control groups [50, 56, 57], and three had usual care control groups [49, 52, 54]. One trial used an attention control design, with the control group offered a limited intervention (written materials only) at the end of the study [53].

#### Intervention characteristics

One intervention was delivered by email [48], one delivered by mail [54, 55], and one delivered using Facebook [56], and all others used a combination of delivery formats, including telephone [49, 50, 52, 53, 57, 58], mail [50, 53], and face-to-face counseling [51–53, 57, 58]. The majority were home-based, with only one intervention reporting supervised PA sessions [51]. Three were walking interventions [51, 52, 58], and four had PA goals that were based on duration [49, 54, 56, 57] and/or moderate intensity [50, 53, 54, 56, 57]. One targeted resistance training [54].

Interventions were commonly 12 weeks in duration [48, 50, 51, 53, 54, 56, 58] and ranged from 6 [52] to 18 weeks [57]. The average number of intervention contacts was 15, and ranged from 1 [54] to 52 [56]. Intervention adherence was high, ranging from 94 % compliance with home exercise logs [58] to 99 % of total contacts completed [51]. Telephone counseling adherence was also high with a median of 9 (of 11) calls completed [49] and a mean of 11 (of 12) calls completed [50, 53]. The intervention delivered using Facebook reported lower adherence, with 81 % of intervention participants who reported receiving ten or more messages from Facebook, and 49 % had made two or more Facebook posts [56].

#### Outcome assessment

Two trials used an objective measure (accelerometer) to assess PA behavior change [50, 51]. All others relied on self-report measures [48, 49, 52–54, 56–58] or used an objective measure in a subsample only [58]. Effect sizes for PA behavior change were reported in four studies (*d* = 0.55–1.93) [51, 53, 54, 57]. Three home-based walking interventions reported significant improvements postintervention (6–12 weeks) to total PA (*d* = 1.02; *P* = 0.004) [51] and walking [52, 58]. Three moderate intensity interventions reported significant postintervention increases in PA (*d* = 0.55; *P* < 0.05) (*d* = 1.93; *P* = 0.02) [50, 53, 57], and two reported nonsignificant increases to aerobic and moderate-vigorous PA [54, 56]. One trial that targeted resistance training reported significant improvements and that the odds of meeting the resistance training guidelines had increased by 3.38 in the tailored intervention group [54].

Of the four trials that reported follow-up assessments of 6 months or longer [51, 53, 57, 68], only two reported behavior change 3 months after intervention completion [53, 68]. One trial reported that accelerometer-assessed behavior was maintained [68], and one reported that there were significant postintervention changes that were not maintained at 6 and 12 month follow-ups [53]. Study retention was high, with a mean retention rate of 86 % (range 71 % [57] to 95 % [50, 53]). One trial [52] reported adverse events involving two participants that experienced anemia, shortness of breath, and dizziness. Participants in this trial were undergoing active treatment at the time of intervention.

### Meta-analysis of SCT intervention effects on physical activity

**Fig. 2:**
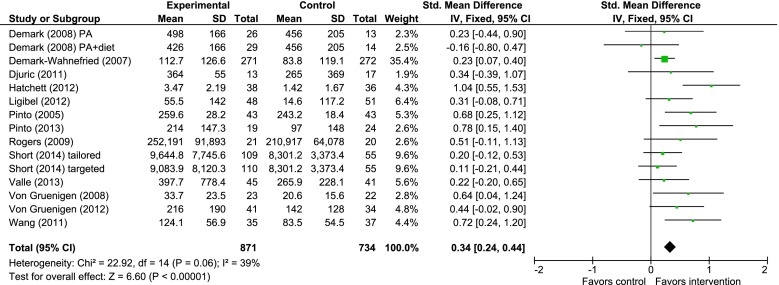
Meta-analysis examining the effects of SCT-based interventions on physical activity immediately postintervention

Meta-analysis was conducted with 12 trials, which reported PA outcomes [48–54, 56–58, 60, 63–65] in Fig. 2. Six trials were not included in the meta-analysis [57–59, 61, 66, 67]. Reasons for exclusion were as follows: diet-only study [59]; did not report adequate information from the modified PA recall [61]; and did not report duration of PA [66]; or had a high risk of bias [57, 58, 67].

Two trials consisted of three study arms, which compared two PA interventions to a standard recommendation control [54, 55], and a PA intervention to a PA and diet intervention, compared to an attention control group [60]. The results for each intervention compared to the control group are reported separately in the meta-analysis. Results were pooled to establish the effects of interventions on total PA at intervention completion. As there was moderate heterogeneity among interventions (*χ* ^2^ = 22.71, *df* = 13 [*P* = 0.05]; *I* ^2^ = 43 %), the random effects models were used. The impact of interventions on PA immediately postintervention was significant (SMD = 0.33 [0.23, 0.44], *Z* = 6.34 [*P* < 0.00001]) (Fig. 2). Sensitivity analysis was undertaken that compared this analysis to a meta-analysis which included the three trials with high risk of bias [57, 58, 67]. There was no change to the impact of interventions on total PA (SMD = 0.34 [0.24, 0.44]). However, there was an increase in heterogeneity (*χ* ^2^ = 30.31, *df* = 16 [*P* = 0.02]; *I* ^2^ = 47 %).

### Diet-only trial

One trial reported dietary outcomes only [59]. The trial targeted men receiving active surveillance for prostate cancer, with a dietary counseling intervention delivered by telephone over 6 months. Men completed self-report measures and provided blood samples for objective assessment of carotenoid intake at completion of the intervention. Retention rate was 97 %, with significant increases to vegetable consumption. There was no change to fruit, whole grains, beans, or fat consumption [59].

### Multiple behavior trials

Seven studies focused on multiple behaviors (PA and diet) [60, 61, 63–66].

#### Participants

A total of 1107 participants were randomized (range 40–543). Three studies recruited newly diagnosed patients [60, 62–64], and patients were diagnosed with breast [60, 62–64, 66], prostate [62, 63], colorectal [61], or endometrial [65, 67, 73] cancer. Only three trials reported time since diagnosis, which varied from a mean of 3.8 months [63], to a mean of 20.6 months [65, 67]. In two trials, participants were scheduled to start chemotherapy [60, 64]. Three trials were aimed exclusively at overweight or obese breast [66] or endometrial cancer survivors [65, 67], with the aim of achieving weight loss through changing PA and diet behaviors. Two trials reported PA [66] or PA and diet [64] outcomes for the purpose of intervention adherence.

Three trials had a usual care control group [65–67], and one used a usual care comparison group with tailored newsletters at study completion [61]. Two had attention control groups [60, 63], and one used attention control with the same written materials and pedometer as the intervention group [64].

#### Intervention characteristics

All of the interventions were home-based and did not include any supervised PA. All trials targeted both PA and diet behaviors. Four of these targeted weight loss [60, 64–67], or prevention of weight gain [60, 64–66], through changing PA and diet behaviors [60, 64–66]. Four trials were aimed at increasing fruit and vegetables and reducing fat [60, 61, 63, 65, 67], one targeted only energy and fat [66], two included both aerobic and strength activity [60, 67], two targeted moderate or moderate-to-vigorous PA [64, 66], and the remaining three targeted PA [61, 63, 65].

Interventions were delivered using telephone counseling [60, 61, 64–67], written materials [60–62, 64, 65, 67], or face to face [65–67]. Most trials used multiple delivery modes, with one that used tailored newsletters [63]. Intervention duration was 6 months [60, 65], 9 months [61], 10 months [62], or 12 months [64, 66, 67]. The average number of contacts was 27 (ranging from 4 [61] to 88 [66]). Studies reported high adherence with all intervention components ranging from 73 to 100 %, with no difference between delivery modes.

#### Outcome assessment

Objectively assessed weight or body fat was reported as the primary outcome in five studies [60, 64–67]. All trials assessed diet using a range of self-reported measures [60, 61, 63–67]. Only one trial used an objective measure of PA (accelerometer) [60], and the remaining trials assessed PA by self-report only [61, 62, 64–67].

Follow-up periods were 6 months in one study [60], 12 months [61, 64–67], or 2 years [62]. However, only two studies reported follow-up beyond postintervention time point [63, 65]. At 12 months, one trial reported significant differences in PA levels, with no difference in diet [65]. After 2 years of follow-up, both study groups had maintained increased fruit and vegetable consumption, decreased saturated fat, and improved overall diet quality [74]. The mean retention rate was 84 % (range 75 % [64] to 96 %[63]). No adverse events were reported in two studies [60, 61, 63, 64]. In two studies that reported adverse events, 10–13 % (*n* = 4; *n* = 74) [63, 64] of the total sample reported serious adverse events that led to withdrawal.

Five of the seven studies [60, 61, 63, 64, 67] reported significant improvements in one or more aspects of diet quality, as assessed by self-report, over the medium to long term (6 months to 2 years). The remaining two studies reported nonsignificant decreases in energy [65, 66] and fat intake in the intervention groups [66]. Inconsistent improvements in fruit and vegetable consumption were reported using a two-item screening question; however, these improvements were not found when using the comprehensive Food Frequency Questionnaire results [61]. At 6 months, significant improvements were reported for vegetables [60], fruit [60], combined fruit and vegetables [67], and decreased fat [60]; however, there was no change for energy [60]. At 12 months, significant improvements were reported for fruit and vegetables, by a mean of 0.5 [63], 0.9 [67] to 3.1 serves per day [64]. There was also a significant reduction by 2.3 % in the proportion of energy from fat [63], which is supported by similar reductions in other trials [64–66]. There were two trials that reported no effect on PA at 3, 6, or 12 months [60, 61]. At 12 months, four trials reported improvements in PA, ranging from 20 min per week (*P* = 0.02) [63] to 89 min per week [67] to 117 min per week [64], and a difference of 17.8 (*P* = 0.002) on the Leisure Score Index [65].

### Theoretical framework

Recent development of behavior change taxonomies [28, 75, 76] has encouraged consistent reporting of behavior change techniques. We have mapped the SCT constructs to identify the behavior change techniques that align with each construct, using the taxonomy designed to change PA and eating behaviors [75] in Table 3.

**Table 3:** SCT constructs mapped to behavior change techniques using the CALO-RE taxonomy [75]

SCT construct	Behavior change technique number	Behavior change technique description
Knowledge	1	Provide information on consequences of behavior in general
	2	Provide information on consequences of behavior to the individual
Self-efficacy	16	Prompt self-monitoring of behavior
	17	Prompt self-monitoring of behavioral outcome
	21	Provide instruction on how to perform the behavior
	22	Model/demonstrate the behavior
	26	Prompt practice
	27	Use of follow-up prompts
Goals	5	Goal setting (behavior)
	6	Goal setting (outcome)
	7	Action planning
	10	Prompt review of behavioral goals
	11	Prompt review of outcome goals
Outcome expectations	16	Prompt self-monitoring of behavior
	17	Prompt self-monitoring of behavioral outcome
	23	Teach to use prompts/cues
	24	Environmental restructuring
	28	Facilitate social comparison
	29	Plan social support/social change
	31	Prompt anticipated regret
	35	Relapse prevention/coping planning
Facilitators/impediments	8	Barrier identification/problem solving
	18	Prompting focus on past success
	29	Plan social support/social change

Self-efficacy was the most commonly assessed construct [48–50, 52, 55–57], with four PA studies that assessed other SCT constructs [48, 51, 53, 55] (Table 4). Two studies [52, 57] reported that the study was based on Bandura’s self-efficacy theory, and the only construct operationalized was self-efficacy. Four PA interventions that used telephone or face-to-face counseling reported that the counseling principles were based on SCT [49, 51, 53, 58]. Five studies reported that the study was based on SCT and the transtheoretical model (TTM), or elements of TTM, such as stages of change [50, 53, 57, 61, 62]. Stage of change was assessed in four studies [50, 51, 53, 57] with reference to both the TTM and social cognitive theories. However, one trial assessed stage of change, despite not providing any reference to TTM or rationale for why stage of change was assessed [51]. Most studies reported using goal setting [48, 51–54, 56–58, 77]; however, few specifically reported action plans [54] or review of goals [56–58]. The most common strategy to increase self-efficacy was to provide a pedometer [49, 50, 52, 53, 56, 57] and/or a log sheet for self-monitoring of PA behavior [49–53]. Social support or social comparison was the most common outcome expectancy targeted [48, 51, 54, 56, 58], and two trials reported strategies targeting environment [51, 54] or relapse prevention [49, 53]. Five trials incorporated identification and discussion of barriers and how to overcome them [50, 51, 53, 57, 58], but only one prompted a focus on past successful strategies [49].

**Table 4:** Social cognitive theory constructs operationalized

Study	Theoretical basis	SCT constructs operationalized	How constructs were operationalized	Constructs measured (no. of items)	Results
PA-only trials					
Short et al. [54, 55]	One intervention group (G2) received computer-tailored newsletters based on SCT G3 (targeted-print) intervention received a Theory of Planned Behavior-based booklet (previously evaluated)	Knowledge of PA guidelines, beneficial outcomes of PA, action planning, feedback on PA performance, social support, role modeling, physical environment	G2: tailored-print newsletters (*n* = 3) tailored using information from individual assessments at baseline, and “update cards” assessing PA and goal setting behavior over the last month. Newsletter 1 strategies were advice for meeting the PA guidelines for cancer survivors, information about the beneficial outcomes of PA, advice on exercising safely, and action planning. Newsletter 2 strategies were expert advice from a behavior change expert, feedback on PA performance, a testimonial, advice on enhancing social support, and action planning. Newsletter 3 contained expert advice from an exercise physiologist, feedback on PA performance, tips on changing the PA environment, information on gaining further support, and action planning	Outcome expectations (11 items); outcome expectancies (1 item); task self-efficacy (7 items); barrier self-efficacy (17 items); behavioral capability (6 items); social support (15 items); perceived built environment (7 items); self-regulation (12 items); action planning (4 items)	Not reported
Valle et al. [56]	SCT with focus on strategies to enhance self-efficacy, behavioral capability, self-monitoring, and social support	Social support, problem solving, self-monitoring, maintaining PA, goal setting, personalized feedback	FITNET intervention goal was to meet PA recommendation for cancer survivors (150 min moderate intensity PA/week). Behavioral capability was operationalized through links to publicly available websites related to PA and/or cancer survivorship, 12 weekly Facebook messages with expanded behavioral lessons on PA topics and behavioral strategies; self-efficacy was operationalized by pedometer which provides feedback on daily walking, website with weekly goal setting and charts providing feedback on performance relative to weekly exercise goal, previous weeks and overall intervention goal; self-monitoring was operationalized with a pedometer to monitor steps, website with diary to record walking steps and PA type, duration, and intensity; and social support was operationalized through the Facebook group with moderated discussion prompts to encourage support, links, and weekly reminders	None reported	
Rogers et al. [51, 68–70]	SCT self-efficacy, emotional coping, reciprocal determinism, perceived barriers, outcome expectations, behavioral capability, goal setting, environment, observational learning, and self-control	Social support, exercise barriers, self-efficacy, goal setting, environment, self-monitoring, barrier self-efficacy, task self-efficacy, barrier interference, outcome expectations, value (outcome importance), enjoyment, fear of exercise, role model, exercise partner	Participants attended 6 discussion group sessions with a clinical psychologist who encouraged social support, provided breast cancer survivor exercise role models, and covered the following topics: journaling, time management, stress management, dealing with exercise barriers, and behavior modification. The specific SCT constructs addressed by the group sessions included self-efficacy, emotional coping, reciprocal determinism, perceived barriers, outcome expectations, behavioral capability, goal setting, environment, observational observational learning, and self-control. Participants also attended 12 individual supervised exercise and 3 individual “face-to-face” update counseling sessions with an exercise specialist that tapered to a home-based program by the end of the intervention. The specific SCT constructs addressed by the individual sessions included self-efficacy, outcome expectations, behavioral capability, perceived barriers, and goal setting with self-monitoring. To further enhance self-monitoring, participants were encouraged to “convert” the minutes spent in PA recorded on their weekly exercise logs into “miles” (i.e., 1 min = 2 miles), which were graphed on a map	PA stage of change (5 items); barrier self-efficacy (9 items); task self-efficacy (4 items); barrier interference (21 items); social support (4 items), positive expectations (14 items); negative outcome expectations (3 items); fear of exercise (1 item); PA enjoyment (1 item); exercise role models (3 items); exercise partner (1 item)	Medium-to-large effect size increase was noted for stage of change (mean difference = 0.95; 95 % CI = 0.75–1.83; *d* = 0.71; *P* = 0.034). Compared with usual care, the intervention group reported lower barriers interference (mean difference = −7.8; *P* = 0.04) and greater PA enjoyment (mean difference = 0.7; *P* = 0.06). Statistically nonsignificant small-to-medium positive effect size increases were noted for barrier self-efficacy, family social support, and total social support, while positive outcome expectations, negative outcome expectations, and negative outcome values demonstrated small-to-medium negative effect size changes for the intervention compared to the usual care group. Little to no change was noted for task self-efficacy, friend social support, importance of positive outcomes, fear of exercise, exercise partner, and role models *Mediation*: Barriers interference mediated 39 % (*P* = 0.004) of the intervention effect on PA 3 months postintervention. PA enjoyment was not a significant mediator. Reducing barriers to PA partially explained intervention effect
Pinto et al. [50, 71, 72]	Intervention based on transtheoretical model (TTM) and SCT. Pinto et al. 2005 [50] state that intervention is based on TTM only	TTM: counseling tailored to participant’s stage of readiness to change, SCT: self-efficacy, goals, PA barriers	Each PA participant received in-person instructions on how to exercise at a moderate intensity level, how to monitor heart rate, and how to warm up before exercise and cool down after exercise. They were given home logs to monitor PA participation and a pedometer. Each participant received a weekly telephone call over 12 weeks from research staff to monitor PA participation, identify relevant health problems, problem solve any barriers to PA, and reinforce participants for their efforts. Finally, a feedback letter summarizing the participant’s progress (e.g., number of PA sessions, average duration of each session, and the participant’s barriers to PA and suggestions to overcome them) was sent to the patient at weeks 2, 4, 8, and 12. At the weekly calls, subjects reported on the PA recorded on home logs, and they received feedback	Decisional balance pros and cons (16 items), exercise self-efficacy (5 items), stage of motivational readiness for PA (4 items)	No significant changes in decisional balance pros, decisional balance cons, or stage of change. Baseline self-efficacy was a significant positive predictor of mean minutes of weekly exercise (*B* = 79.46 min; *P* = 0.004), mean pedometer steps per week (*B* = 2636.9 steps; *P* = 0.0006)
Bennett et al. [57]	TTM and perceived self-efficacy from SCT	Self-efficacy, goals	During the initial counseling session, the participant was encouraged to identify barriers to engaging in regular exercise, and the PA counselor and the participant worked together to develop ideas to overcome barriers. A goal of 30 min of moderate intensity planned PA on most days of the week, but some participants started with more modest goals. Each intervention participant received a pedometer and was shown how to use it as a motivator for walking exercise, but participants were not required to walk if they preferred another form of moderate intensity exercise. Telephone calls were planned to last about 20 min, and the conversation included motivational strategies directed at solving problems, offering encouragement, and reformulating goals, if needed	Self-efficacy for regular PA (6 items); stage of change for exercise (6 items: baseline only)	Self-efficacy was tested as a moderator of intervention effects. Individuals with high self-efficacy in the intervention increased PA levels faster over 6 months than low self-efficacy individuals in the intervention group. In the control group, self-efficacy had no impact on PA levels (*B* = 121.35; *P* < 0.05)
Matthews et al. [58]	Structured behavioral counseling grounded in SCT (using semistructured script)	Goals, PA enjoyment, positive reinforcement, self-reward, personal motivation, barriers, problem solving, social support, goal review, self-efficacy, self-monitoring	The initial counseling session emphasized goal setting and PA safety. Subsequent counseling calls were designed to monitor participant safety and enhance adherence through structured behavioral counseling that was grounded in SCT. A semistructured script was used by the counselors in each of the calls to initiate discussion with participants about their experience in meeting (or not) their walking goals that were agreed upon at the previous intervention contact. Taking their cues from the information provided by the participants in these conversations, the staff then delivered appropriate intervention messages. When participants met their goals, individualized positive reinforcement was provided in the form of a discussion of enjoyment associated with being active and relevant self-rewards. Discussion of personal motivations that helped the individual meet their walking goals was also emphasized. In contrast, if the participant did not meet their walking goals, the conversation naturally led to the barriers participants experienced in the period, and the counselor initiated a conversation about problem solving strategies that might help overcome anticipated barriers in the coming week(s). When appropriate, participants were encouraged to elicit social support from their family and friends that might help them meet their goals (e.g., a walking partner, help with other time commitments). Calls were ended with a recap of the conversation (by the counselor) that included a review of the agreed upon goal for the next week(s), a review of the behavioral issues that were discussed during the call (e.g., positive reinforcements or barriers/problem solving), and an indication of when the next counseling call would occur	None	
Ligibel et al. [49]	SCT and client-centered counseling	Goal setting, self-efficacy, self-monitoring	Initial calls focused on goal setting and performance assessment so as to build self-efficacy for exercise behaviors, while later calls concentrated upon the adequacy of plans for relapse prevention. Each call reviewed performance on the behaviors previously discussed and encouraged the participant to keep using self-regulatory skills to achieve change. The telephone calls were supplemented by a Participant Workbook, which included additional information regarding the importance of exercise in cancer populations, guidelines for exercise safety, and journal pages to track weekly exercise. Participants were provided with a pedometer. Instructions for using the pedometer were included in the Participant Workbook and were reviewed during the first counseling session. Participants were asked to record the number of minutes of exercise they performed and steps they completed each day in journals, which were reviewed during the telephone counseling calls	Self-efficacy (5 items)	Intervention participants reported trends toward improvement in exercise self-efficacy (0.1 ± 1.2 vs −0.3 ( ± 0.8) (*P* = 0.06), as compared with controls
Wang et al. [52]	Bandura’s self-efficacy theory	Self-efficacy	Discuss program with women and make their own weekly walking goal for exercise; encourage women to document weekly walking logs so they can see their own progress during the program; story telling/role model story in booklet; the researcher will make weekly phone calls to understand women’s feelings, the effects, and the countereffects of exercise, and will praise women’s performance and encourage women to keep progressing in the program for their personal goals; self-monitoring with the heart rate ring and pedometer during exercise; introduce the walking program with written material and verbal explanation by the researcher including warm up, cool down, and progressively increasing intensity, frequency, and duration over time	Exercise self-efficacy scale (18 items)	Subjects in the exercise group had significantly better exercise self-efficacy than those in the usual care group over the intervention period. At baseline, the intervention group was +13.5 points higher, and at time 4, the difference had increased to +31.3 (*P* < 0.001)
Pinto et al. [53]	Transtheoretical model and the SCT	Self-efficacy, outcome expectations, stimulus control, reinforcement management, self-monitoring, goals, planning	Participants received in-person instructions on how to exercise at a moderate intensity level, how to monitor heart rate, and how to warm up before exercise and cool down after exercise. They were given home logs to monitor PA participation and a pedometer. Each participant received a weekly call over 12 weeks from research staff to monitor PA participation, identify relevant health problems, problem solve any barriers to PA, and reinforce participants for their efforts. Activity counseling was based on the transtheoretical model and the social cognitive theory and tailored to each participant’s motivational readiness. The counseling focused on strengthening self-efficacy for exercise, on setting realistic outcome expectations, and on training participants in using behavioral processes of change such as stimulus control and reinforcement management and in using techniques such as self-monitoring of exercise behavior, setting exercise goals, and planning for exercise. After the 12 week program was completed, monthly phone calls were provided for 3 months to reinforce progress, identify lapses from PA, and recover from any lapses that may have occurred. Finally, a feedback letter summarizing participants’ progress was sent at weeks 2, 4, 8, and 12	Stage of motivational readiness for PA (5 items)	The intervention produced strong effects on participants’ motivational readiness at 3 months (OR = 5.26, 95 % CI = 1.32–20.93; *P* = 0.018) that were attenuated at 6 months (OR = 3.81, 95 % CI = 0.90–16.71; *P* = 0.070) and weakened further at 12 months (OR = 1.89, 95 % CI = 0.52–6.86; *P* = 0.335)
Hatchett et al. [48]	SCT	Self-efficacy, goal setting, anticipated result of exercise, time management, self-monitoring, barriers, relapse prevention	The e-counselor offered advice regarding exercise and PA. The researchers believed that if a participant were asked to offer information regarding her behavior during the intervention, she would be more likely to engage in the desired behavior. The topics of each email are as follows: week 1: goal setting, anticipated result of exercise; week 2: goal setting, time management, self-monitoring; week 3: self-monitoring, description of an exerciser, overcoming barriers; week 4: self-monitoring, barriers to exercise; week 5: self-monitoring, overcoming barriers, describe the anticipated outcomes of exercise; week 7: goal setting, self-monitoring, time management, relapse prevention; week 9: overcoming barriers, goal setting, self-monitoring, time management, relapse prevention; week 11: properties of an exerciser, results of cancer	SCT variables: self-regulation (20 items); outcome expectancy values (19 items); exercise self-efficacy (14 items); exercise role identity (9 items)	Not reported
Diet-only trials					
Parsons et al. [59]	Strategies adopted from SCT	Not described	The principle strategy to promote dietary change in the intervention arm was a telephone counseling protocol with individualized, direct assistance tailored to each participant. The telephone counseling protocol followed a stepwise, phased approach that used strategies adopted from SCT. Motivational interviewing techniques were used to help participants assume and maintain responsibility for their behavioral change. No other details reported	Not reported	
Multiple behavior trials					
Demark-Wahnefried et al.—STRENGTH [60]	SCT (key concepts of promoting self-efficacy and behavioral monitoring)	Self-efficacy, behavioral monitoring	Written and verbal instruction based on SCT (key concepts of promoting self-efficacy and behavioral monitoring) (a workbook and telephone counseling). No other details reported	Confidence (self-efficacy) in making changes in their dietary or exercise practices (did not specify number of items)	Not reported
Campbell et al. [61, 111, 112]	TTM and SCT	Stages of change, social support, barriers to change, knowledge, role models, self-efficacy	G2 received tailored- print expert feedback driven by baseline data. G3 received motivational interviewing telephone calls that encouraged participants to overcome ambivalence and identify their own strategies for change. G4 received both the tailored-print feedback and motivational interviewing telephone calls	Self-efficacy—eating fruit and vegetables, and engaging in PA (2 items). Social support for healthy eating and exercise (4 items). Perceived barriers to behavior change (6 items), knowledge of recommendations (1 item)	*Mediation*: None mediated dietary change. Higher self-efficacy was associated with greater fruit and vegetable consumption at both baseline and follow-up, but increase in self-efficacy did not predict greater change in fruit and vegetable consumption There were no intervention effects for colorectal cancer survivors
Von Gruenigen et al. [65, 73]	SCT	Establish short-term goals, build self-efficacy, reinforcement, individual progress toward goals, emphasis on long-term change, patient feedback	The protocol followed a stepwise, phased approach using strategies outlined by SCT, indicating that the optimal intervention for a major behavior change should focus on establishing short-term goals, and enabling the person to build self-efficacy. Participants were contacted by the research dietician by phone or newsletter every week that the group did not meet. Phone calls were structured in content and included reinforcement and discussion regarding the previous week’s topic. Participants were also given feedback on individual progress toward PA and nutrition goals. Newsletter topics included the following: holiday recipes, reinforcement of nutrition goals, ways to increase PA and step count, restaurant menu makeovers, and eating on the run	Self-efficacy using the Weight Efficacy Life-Style (WEL) questionnaire (20 items). Self-efficacy specific to eating behaviors in five situational factors: negative emotions, food availability, social pressure, physical discomfort, and positive activities	Significant difference in “social pressure” subscale (*P* = 0.03). Increase in self-efficacy related to negative emotions (*P* < 0.01), food availability (*P* = 0.03), and physical discomfort (*P* = 0.01) in those women who lost weight during the year. At 12 months, self-efficacy scores remained high (6 months after intervention had concluded). Morbidly obese patients had significantly decreased self-efficacy when feeling physical discomfort and decreased total self-efficacy score. There was a significant effect for self-efficacy related to social pressure and restraint improved. For self-efficacy related to negative emotions, there was a mean increase of 8.9 in women who lost weight versus 0.6 in those whose weight was stable or who had gained weight
Von Gruenigen et al. [67]	SCT with a focus on establishing short-term goals, enabling the person to build self-efficacy	The intervention followed a stepwise, phased approach with a focus on establishing short-term goals, enabling the person to build self-efficacy	Individual expert physician counseling, individual goal setting, goal reinforcement in newsletters, social support and eating in social situations, planning meals and grocery shopping, how to read food labels, pedometers provided feedback and reinforcement of PA goals. Incremental goals (for months 1–2, months 5–6), modeling of resistance exercise. The intervention focused on the adoption of lifelong changes rather than caloric restriction. Education and skill development to increase PA and PA self-efficacy were included using a guide previously developed for breast cancer survivors. Patients were encouraged to add activities that they enjoyed or to begin a walking program or other exercise activity. Long-term changes in everyday activities (for example, climbing stairs instead of taking elevators) and moderate aerobic activity were emphasized. Participants were given pedometers to provide immediate feedback and reinforcement to patients and to provide objective assessment of PA. Patients were given 3 lb hand and adjustable ankle weights and instructed in the proper form and procedure for performing resistance exercises. Heart rate monitors were provided to facilitate monitoring of target heart rate goals. Physician counseling visits (conducted by the PI) at 3, 6, and 12 months focused on nutrition and PA goals for SUCCEED participants in order to augment the group sessions and provide individualized attention	Not reported	
Demark-Wahnefried et al.—FRESH START [62, 63, 78]	SCT: cues to action, self-efficacy, skill development, goals, goal reinforcement. Messages were customized to stages of change (TTM)	Benchmark behavior, goal, behavior logs, behavioral cues, tailored to stages of change, goal, testimonial, overcoming barriers, benefits, progress to goal	The FRESH START intervention was based on the SCT that emphasizes confidence building and skills development; the transtheoretical model also was used to frame messages on participants’ stage of readiness to motivate behavior change. Participants are encouraged to set small incremental goals, which, when achieved, are reinforced to build self-efficacy. To build upon self-efficacy incrementally, participants are assisted in making changes in one behavioral domain at a time. Participants are first assigned the behavior with the highest self-efficacy score, and behaviors with lower scores are presented subsequently (with the premise that after the participant achieves successful behavior change in the first area, he or she can generalize this success to the next health domain). In situations where self-efficacy scores are equal for the two behaviors, the most advanced stage of readiness will dictate the first domain targeted. For participants reporting 3 deficient behavioral areas, the initial intervention materials target the behavior associated with the highest self-efficacy score, and the second behavioral area is selected at random. In the initial mailing, participants receive a personalized workbook that includes the first unit materials, and a second installment of workbook materials arrives midway through the intervention. For each unit, the first page is a feedback form in which the participant’s behavior is compared with goal behavior, and encouragement is provided to achieve the goal. Each installment of the workbook includes personalized behavior record logs that correspond to the content areas to help participants track behavior (to promote change and improve self-reporting accuracy). In addition, each installment of the workbook includes items that serve as behavioral cues [i.e., a pedometer and Therabands® accompany the exercise unit]. Newsletters are 4 pages of colorful graphics and text that include the following components: (1) a personalized greeting tailored to stage of readiness; (2) a goal statement that reflects engagement in goal setting behavior; (3) a testimonial tailored on age, race, and cancer coping style; (4) an advice column that provides guidance for overcoming barriers—tailored to a subject’s reported barriers; (5) a “Fun Facts” section—untailored; (6) a benefits section that is untailored and emphasizes the benefits of practicing goal behavior; (7) a status section that features a graph depicting the participant’s progress in relation to goal and accompanying tailored messages [i.e., achievement of goal (praise), progress toward goal (praise and encouragement), no progress (encouragement), or the absence of data (encouragement to submit updated data)]	Self-efficacy (PA and diet) (3 items), stage of readiness (range 3–12 items, depending on responses), social support (11 items), barriers (37 items)	The intervention was not significantly associated with self-efficacy for exercise; however, there was a positive correlation obtained between self-efficacy for exercise and total minutes per week of exercise at follow-up *Mediation*: Results support the hypothesis that changes in self-efficacy for fat restriction and eating more fruit and vegetables partially mediate the effects of the intervention on diet quality (37.7 % variance, *P* < 0.001). Furthermore, change in self-efficacy for fat restriction partially mediated the intervention’s effects on the percentage of kilocalories from fat (30.1 % variance, *P* < 0.001), and change in self-efficacy for fruit and vegetable consumption partially mediated the intervention’s effects on daily servings of fruit and vegetables
Djuric et al. [64]	SCT—the telephone counseling approach blended motivational interviewing (MI) with SCT	Goals, self-monitoring, self-efficacy	The telephone counseling approach blended MI with SCT. They also received pedometers, a daily food and exercise log, and example menus at individually appropriate calorie levels. The counseling plan was for the dietician to contact subjects weekly for the first two calls, biweekly for the next 5 months, and monthly for the last 6 months, for a total of 19 calls. The self-monitoring logs were reviewed during the calls. The counseling approach combined principles of SCT and MI. Subjects were involved in deriving their own short-term goals and evaluating their progress toward goals. To build self-efficacy, any positive changes on the self-monitoring sheets were identified and praised	Self-efficacy (6 items), self-confidence for maintaining a healthy lifestyle (6 items)	Not reported
Djuric et al. [66]	SCT	Self-monitoring, goal setting, self-efficacy, consideration of body image, social support, removing roadblocks, positive thinking, dealing with high-risk situations and slips, and cue elimination	G3 (individualized arm): Monthly written information was prepared on various weight loss topics (environmental control, serving size control, exercise, motivation, goal setting, holiday eating, seasonal foods) and either presented to the women at the monthly meeting or mailed to their homes. Pedometers were provided for self-monitoring and goal setting. It was requested that exercise and dietary logs be kept daily, and these were reviewed together with each subject. Contacts were by phone or in person, and food and exercise records were mailed to the dietician before the scheduled contact. The counseling session varied in length depending on individual needs. The dietician first verified whether or not the participant was meeting behavior change goals set in the previous week. If not, the problem was delineated, and the dietician helped the subject devise a plan that would be used to circumvent the problem. The techniques taught included goal setting, menu planning, self-efficacy, self-monitoring, consideration of body image, social support, social eating, removing roadblocks, positive thinking, dealing with high-risk situations and slips, and cue elimination G4 (comprehensive arm): Subjects received the individualized counseling described above and were asked to attend weekly weight watchers meetings using free coupons	None reported	Not reported

In PA-only trials, improvements in self-efficacy were associated with increased PA in three studies [49, 50, 52]. Moderation analyses identified that intervention participants with high self-efficacy increased their PA levels faster over the 6 month assessment period compared to intervention participants with low self-efficacy [57]. Mediation analyses identified that improvements in barrier interference and barrier self-efficacy mediated 39 and 19 % of the intervention effect on PA maintenance 3 months after the intervention [70]. There were no significant changes in decisional balance pros, cons, or experiential processes of change [71, 72], or task self-efficacy, social support, outcome expectations, or fear of exercise [70]. Two trials assessed but did not report results for self-regulation, outcome expectancy values, exercise self-efficacy, exercise role identity, behavioral capability, or social support [48, 54, 55]. Intervention effects on stage of change results were mixed, with one trial that reported a medium-to-large effect [51], one reported significant postintervention improvements that declined over subsequent follow-ups [53], and one reported no effect on stage of change [71].

The diet-only trial reported that the telephone counseling protocol “used strategies adopted from SCT”; however, no further detail was provided [59]. In multiple behavior studies, one did not report how SCT constructs were operationalized [60]. All other trials reported goal setting, self-monitoring, building self-efficacy (for PA and diet) [60–63, 67], or diet [65, 73], or for maintaining a healthy lifestyle [64], overcoming barriers, and social support [61–66, 73]. Goal setting [48, 63–67] and review of goals [63–66] were commonly operationalized. Self-monitoring was commonly operationalized through providing a pedometer [63, 64, 66, 67] or log sheet [63, 64, 66]. Few trials reported how they operationalized outcome expectations, with only three that reported social support [63, 66, 67], one that included environment [66], and one that reported relapse prevention [48]. Four studies included identification of barriers and how to overcome them [48, 61, 63, 66]. Self-efficacy did not appear to be related to PA behavior change [60, 63]. Improvements to diet quality were partially mediated by changes in self-efficacy for fat restriction and eating more fruit and vegetables [62, 63, 78]. Only the social pressure subscale of self-efficacy was significantly related to eating behaviors (*P* = 0.03) [65, 73]. Two trials reported that self-efficacy was not associated with diet changes [60] or fruit and vegetable consumption [61]. Social support for healthy eating, perceived barriers to behavior change, and knowledge of recommendations were assessed, but none mediated fruit and vegetable consumption [61].

## Discussion

### Overview of findings

The aims of this review were to synthesize the existing literature relating to PA and diet interventions based on SCT that target cancer survivors and to identify successful strategies to assist cancer survivors in making positive PA and diet behavior change. This review supports the efficacy of SCT-based interventions in changing PA and diet behavior in cancer survivors. Our effect size of 0.33 for PA interventions can be defined as a small-to-medium effect [79] and is similar to other meta-analyses [5, 80, 81] that reported effect sizes of 0.32–0.38 for PA interventions (including cancer survivors both during and after treatment). Our positive results for PA behavior mirror the results reported in two recent reviews examining PA behavior change in breast cancer survivors and also found that trials were mostly PA only, few included objective measures, and few reported postintervention maintenance [82, 83]. While evidence has been building to support the effects of PA and diet behavior on health outcomes, there remains a need to focus on behavior change trials to understand how to promote sustainable healthy behaviors.

Our findings that the majority of included trials reported statistically significant improvements to at least one aspect of diet quality and weight loss are supported by other reviews with the general adult population [80, 81]. Due to considerable heterogeneity in the dietary outcomes assessed, it was not possible to conduct a meta-analysis, although self-reported improvements to diet quality were evident in six of eight studies. The two trials that did not find improvements to dietary quality had a primary aim of decreasing weight, rather than behavior change [65, 66]. More evidence is required from behavior change trials that have an emphasis on dietary change rather than weight.

The limited number of trials and the heterogeneity of included studies in this review prevented any formal subgroup analyses in our review. The effect appeared strongest for PA-only interventions compared to multiple behavior interventions; however, this should be interpreted with caution as PA-only interventions included smaller sample sizes and shorter follow-up periods. A review of single compared to multiple behavior interventions in older adults also reported that PA effects appeared strongest in single behavior change interventions. However, there were inadequate multiple health behavior change interventions to compare to [84]. Similar reviews [80, 81] examined intervention setting, duration, person delivering the intervention, delivery mode, age of target group, and intervention effectiveness and found that only increased contact frequency was associated with increased PA and diet behavior change [80].

### Social cognitive theory

Few trials conducted mediation analyses or reported changes in theoretical constructs. In those that did report the impact of interventions on theoretical constructs, results were inconsistent. Self-efficacy was the only construct that appeared to be associated with positive behavior change for both PA and diet [52, 57, 61–63, 65, 73]; however, mediation analyses in two trials identified that theoretical constructs only partially mediated the intervention effects [62, 63, 70]. Other reviews examining individual SCT constructs have concluded positive outcome expectations, and intentions are associated with behavior change [85, 86]. Self-efficacy and goal setting were commonly operationalized, but there was limited reporting of how other constructs were operationalized as part of the intervention. Recent reviews identified that self-efficacy, self-monitoring of behavior, prompting intention formation, planning, specific goal setting and review, and feedback on performance were associated with increased effectiveness in PA and diet behavior change [80, 81, 87]. However, given the crossover between theoretical constructs and behavior change techniques, the positive results for SCT-based trials in this review may be a result of the individual behavior change techniques employed, such as self-regulatory behaviors, rather than the theoretical constructs.

Recent reviews have questioned the value of theory in developing and evaluating interventions, with two recent behavior change reviews concluding that interventions based on theory were no more effective than atheoretical interventions [80, 88] and another two reviews that supported the efficacy of theory-based interventions [89, 90]. The conflicting results may be due, at least in part, to the inadequate description of how theory is implemented and evaluated in interventions and also due to the overlap with specific behavior change techniques, which have been associated with intervention effectiveness.

There were differences in the risk of bias assessment. The majority of PA-only studies received a strong global rating (*n* = 5), and multiple behavior trials received a moderate global rating (*n* = 5). Trials received a weak global rating because fewer than 60 % of potentially eligible participants agreed to take part before randomization, which is used as an estimate of the external validity of the study [47, 91], and because both the outcome assessor and study participants were aware of the research question [47]. Unlike in clinical trials where participants are unaware of their exposure status, behavior change trials present significant problems with blinding and recruitment as participants are expected to actively engage with the intervention. Self-selection bias is a likely issue in behavior change trials.

### Strengths and weaknesses of review methods

Although this is a comprehensive review of the published literature, there are some limitations that should be noted. Search results were screened for eligibility by only one reviewer, despite recommendations that this step is conducted by two independent reviewers [92]. This review comprehensively searched a number of databases; however, it made no attempt to search for non-English publications or unpublished literature. Potentially eligible study protocols were obtained; however, no attempt was made to contact trial authors to obtain unpublished results of these studies [93–95]. The review included a broad definition of cancer survivors, including those both during and after completion of active treatment. While this increases the breadth of evidence, it likely contributed to the heterogeneity of the included studies.

There are a number of SCT-based health outcomes trials, including the WINS [96–98] and WHEL [99, 100] trials, Active for Life trial [101], and RENEW trial [102, 103], that initially met criteria for inclusion in the review [104]. However, the study team agreed not to include these trials as behavior change was either not reported [101, 104] or reported as a secondary outcome only [96, 99, 102]. Due to the heterogeneity of these trials and the inconsistency in reporting behavior change outcomes, these trials were not included in the review. The definition of SCT-based intervention was relatively broad and not limited by how well SCT was described or operationalized in the intervention. Studies needed to explicitly state that the intervention was based on SCT, which may or may not have included other theories, and was dependent on the author description of the trial. Recent publications have detailed a checklist for evaluating the extent to which an intervention is theory-based [105], which will enable greater clarity in the role of theory in the development of an intervention. There was one trial where it was unclear if the study was based on a theoretical framework. One paper [50] had been screened and judged not eligible due to not reporting that the trial was based on SCT. However, subsequent eligible and included trial papers [71, 72] reported that the intervention was based on SCT, and therefore, this trial was included in the review. It may be that due to publication size restrictions, authors have limited space to fully describe intervention development. Alternatively, theoretical frameworks may be applied post hoc to an intervention. With journals requiring adherence to CONSORT [91], this is likely to improve the consistent and transparent reporting of RCTs.

### Limitations of the included trials

Studies demonstrated moderate heterogeneity, although most interventions included breast cancer patients and were conducted with patients after completion of active treatment. This limits the generalizability of findings to males, to survivors of other cancer types, and to patients undergoing active treatment. Most studies involved small sample sizes and only four trials reported a sample size greater than 100 [49, 54, 61, 63]. Only three of the trials included in this review focused on promoting resistance training [54, 60, 67], despite a recent review and meta-analysis that concluded resistance training has benefits on body composition and muscle strength in cancer patients during and after cancer treatment [106]. Meta-analysis used objectively assessed data, where available [51, 60]; however, the majority of data was self-reported. PA and diet outcomes were predominantly based on self-report data, while weight was frequently measured or objectively assessed [59–62, 64–66]. Participants were not blinded to intervention aims in any trials, so there may be inherent differences between those participants recruited for PA-only interventions, compared to participants interested in diet, weight control, or multiple behavior interventions.

### Future research

This review supports the efficacy of SCT-based interventions in changing PA and diet behavior in cancer survivors. While interventions reported a theoretical basis, these constructs were often inadequately operationalized or reported and rarely measured or tested [89, 107, 108]. Despite a large body of cross-sectional data [33–38] linking SCT constructs with diet and PA behavior, there remains a need to test whether changes in these constructs predict behavior change in interventions. Comparison between health theories would also be a useful gap to address.

Development of the taxonomy assessing the extent to which interventions are theory-based and use of behavior change techniques [81, 105] will contribute evidence to help researchers understand the intervention components that are essential to behavior change. Whether these are related to specific theoretical constructs, or to behavior change techniques, such as self-regulatory techniques, requires further research. Research assessing whether single or multiple health behavior interventions have the greatest benefit to improve PA and diet behaviors is required.

There is a large evidence base supporting the efficacy of PA interventions, and these are predominantly based on breast cancer patients, using short-term, self-reported outcomes. Future studies need to consider how to translate this research into ongoing support and programs to assist cancer survivors to increase and maintain PA levels. Further work should also include trials which focus on resistance training, as there are specific guidelines for cancer survivors to undertake resistance training. The field of dietary interventions is much less developed, with interventions demonstrating that cancer survivors are willing and able to make improvements to diet [13, 63, 80, 99, 102]. As diet quality is comprised of a complex set of behaviors, there is a need to examine the co-occurrence of changes in different aspects of diet [109]. Future studies would benefit from considering the impact of behavior change from a healthy lifestyle perspective, such as considering compliance with World Cancer Research Fund guidelines [14].

### Implications

Despite the limitations of this review, it appears that SCT-based interventions demonstrate promise for improving the PA and diet behaviors of cancer survivors. Interventions using a range of delivery modes all demonstrated significant PA improvements, with a small-to-medium effect size, after a relatively short intervention period (12 weeks). Diet and multiple behavior component interventions tended to have a higher number of intervention contacts and greater intervention duration (6 months). However, the increased contact time did not appear to be related to the magnitude of change. High trial retention across both single and multiple behavior change trials may be related to the low burden of predominantly unsupervised interventions or that cancer survivors are motivated to improve their PA and diet behaviors. Unfortunately, there was little evidence to guide researchers in helping cancer survivors to maintain health behaviors after completion of interventions, and this has been noted previously [83, 110]. Improved description and reporting of intervention content and the way in which theory-based interventions use theory to guide the trial and intervention components remain necessary to understand what factors are driving the results of theory-based interventions. Given the recent mixed findings on the efficacy of theory-based interventions, a greater understanding of how theory is operationalized is necessary to understand what factors contribute to the success of interventions. Further evidence on theory-based trials is required to understand the crossover between theory-based constructs and behavior change techniques and understand the impact of each on improving health behaviors. Research expanding the rigorous implementation and reporting of behavior change techniques is likely to improve understanding of the working mechanisms that underpin how and why an intervention works or does not work.

## Conclusions

SCT-based interventions appear effective in improving PA and diet behaviors. No specific intervention characteristics or theoretical constructs were associated with effectiveness. Future SCT-based interventions should describe the extent to which theoretical or behavior change constructs are implemented and evaluated, in order to identify the successful components of SCT-based interventions.

## Appendix 1: Medline search

1. cancer survivor*.mp.
2. Survivors/
3. cancer*.mp.
4. exp Cancer/
5. 3 or 4
6. 2 and 5
7. cancer patient*.mp.
8. patient*.mp.
9. exp Patients/
10. 8 or 9
11. 5 and 10
12. 1 or 6 or 7 or 11
13. exp Nutrition Surveys/ or exp Nutrition Policy/ or exp Nutrition Assessment/ or exp Nutrition Therapy/
14. Diet, Cariogenic/ or Diet Surveys/ or Diet, Carbohydrate-Restricted/ or Diet Therapy/ or Diet, Atherogenic/ or Diet/ or Diet, Sodium-Restricted/ or Diet, Gluten-Free/ or Diet, Reducing/ or Diet, Fat-Restricted/ or Ketogenic Diet/ or Diet Fads/ or Diet, Macrobiotic/ or Diet, Protein-Restricted/ or Diet, Vegetarian/ or Diet, Mediterranean/ or Diabetic Diet/ or Diet Records/
15. Food Habits/ or exp Food/ or Health Food/ or Food Preferences/
16. 13 or 14 or 15
17. physical activity.mp. or Motor Activity/
18. exercise therapy/ or muscle stretching exercises/ or resistance training/ or strength training
19. Exercise/
20. weight.mp.
21. aerobic.mp. or Physical Exertion/
22. running/ or swimming/ or walking/
23. 17 or 18 or 19 or 20 or 21 or 22
24. 16 or 23
25. Health Behavior/ or Self Efficacy/ or social cognitive theory.mp. or Psychological Theory/ or Social Support/
26. social cognitive.mp.
27. Motivation/ or Health Education/ or Health Promotion/ or social learning theory.mp.
28. 25 or 26 or 27
29. 12 and 24 and 28

## References

[1] Campo RA, Rowland JH, Irwin ML, Nathan PC, Gritz ER, Kinney AY (2011). Cancer prevention after cancer: changing the paradigm—a report from the American Society of Preventive Oncology. Cancer Epidemiol Biomarkers Prev.

[2] Jemal A, Bray F, Center MM, Ferlay J, Ward E, Forman D (2011). Global cancer statistics. CA Cancer J Clin.

[3] Carmack CL, Basen-Engquist K, Gritz ER (2011). Survivors at higher risk for adverse late outcomes due to psychosocial and behavioral risk factors. Cancer Epidemiol Biomarkers Prev.

[4] Cramp F, Daniel J. Exercise for the management of cancer-related fatigue in adults (review). Cochrane Database of Systematic Reviews. 2008;CD006145. 10.1002/14651858.CD006145.pub2 18425939

[5] Speck RR, Courneya KS, Masse LC, Duval S, Schmitz KH (2010). An update of controlled physical activity trials in cancer survivors: a systematic review and meta-analysis. J Cancer Surviv.

[6] Ferrer RA, Huedo-Medina TB, Johnson BT, Ryan S, Pescatello LS (2011). Exercise interventions for cancer survivors: a meta-analysis of quality of life outcomes. Ann Behav Med.

[7] McNeely ML, Campbell KL, Rowe BH, Klassen TP, Mackey JR, Courneya KS (2006). Effects of exercise on breast cancer patients and survivors: a systematic review and meta-analysis. Can Med Assoc J.

[8] Schmitz KH, Courneya KS, Matthews C, Demark-Wahnefried W, Galvao DA, Pinto BM (2010). American College of Sports Medicine roundtable on exercise guidelines for cancer survivors. Med Sci Sport Exerc.

[9] Rock CL, Doyle C, Demark-Wahnefried W, Meyerhardt J, Courneya KS, Schwartz AL (2012). Nutrition and physical activity guidelines for cancer survivors. CA Cancer J Clin.

[10] Ballard-Barbash R, Friedenreich CM, Courneya KS, Siddiqi SM, McTiernan A, Alfano CM (2012). Physical activity, biomarkers, and disease outcomes in cancer survivors: a systematic review. J Natl Cancer Inst.

[11] Demark-Wahnefried W, Clipp EC, Morey MC, Pieper C, Sloane R, Snyder DC (2006). Lifestyle intervention development study to improve physical function in older adults with cancer: outcomes from Project LEAD. J Clin Oncol.

[12] Scheier MF, Helgeson VS, Schulz R, Colvin S, Berga S, Bridges MW (2005). Interventions to enhance physical and psychological functioning among younger women who are ending nonhormonal adjuvant treatment for early-stage breast cancer. J Clin Oncol.

[13] Chlebowski R (2007). Lifestyle change including dietary fat reduction and breast cancer outcome. J Nutr.

[14] World Cancer Research Fund/American Institute for Cancer Research (2007). Food, nutrition, physical activity, and the prevention of cancer: a global perspective.

[15] Psaltopoulou T, Ilias I, Alevizaki M (2010). The role of diet and lifestyle in primary, secondary, and tertiary diabetes prevention: a review of meta-analyses. Rev Diabet Stud.

[16] Hussain A, Claussen B, Ramachandran A, Williams R (2007). Prevention of type 2 diabetes: a review. Diabetes Res Clin Pract.

[17] Artinian NT, Fletcher GF, Mozaffarian D, Kris-Etherton P, Van Horn L, Lichtenstein AH (2010). Interventions to promote physical activity and dietary lifestyle changes for cardiovascular risk factor reduction in adults: a scientific statement from the American Heart Association. Circulation.

[18] Robien K, Demark-Wahnefried W, Rock CL (2011). Evidence-based nutrition guidelines for cancer survivors: current guidelines, knowledge gaps, and future research directions. J Am Diet Assoc.

[19] Hayes SC, Spence RR, Galvão DA, Newton RU (2009). Australian association for exercise and sport science position stand: optimising cancer outcomes through exercise. J Sci Med Sport.

[20] Campbell A, Stevinson C, Crank H (2012). The BASES expert statement on exercise and cancer survivorship. J Sports Sci.

[21] Cancer Council Australia. Position statement on benefits of healthy diet and physical activity for cancer survivors. 2013 Available from: www.cancer.org.au/content/pdf/CancerControlPolicy/PositionStatements/PSbenefitshealthydietcancersurvivorsJUN06.pdf. Accessed 11 Aug 2013.

[22] Courneya KS, Friedenreich CM (2007). Physical activity and cancer control. Semin Oncol Nurs.

[23] Bellizzi KM, Rowland JH, Jeffery DD, McNeel T (2005). Health behaviors of cancer survivors: examining opportunities for cancer control intervention. J Clin Oncol.

[24] Eakin EG, Youlden DR, Baade PD, Lawler SP, Reeves MM, Heyworth JS (2007). Health behaviors of cancer survivors: data from an Australian population-based survey. Cancer Causes Control.

[25] Blanchard CM, Courneya KS, Stein K (2008). Cancer survivors’ adherence to lifestyle behavior recommendations and associations with health-related quality of life: results from the American Cancer Society’s SCS-II. J Clin Oncol.

[26] Glanz K, Bishop DB (2010). The role of behavioral science theory in development and implementation of public health interventions. Annu Rev Public Health.

[27] Noar SM, Benac CN, Harris MS (2007). Does tailoring matter? Meta-analytic review of tailored print health behavior change interventions. Psychol Bull.

[28] Abraham C, Michie S (2008). A taxonomy of behavior change techniques used in interventions. Health Psychol.

[29] Nigg CR, Allegrante JP, Ory M (2002). Theory-comparison and multiple-behavior research: common themes advancing health behavior research. Health Educ Res.

[30] Lubans DR, Foster C, Biddle SJH (2008). A review of mediators of behavior in interventions to promote physical activity among children and adolescents. Prev Med.

[31] Bandura A (2004). Health promotion by social cognitive means. Health Educ Behav.

[32] Bandura A (1998). Health promotion from the perspective of social cognitive theory. Psychol Health.

[33] White S, Wojcicki T, McAuley E (2011). Social cognitive influences on physical activity behavior in middle-aged and older adults. Gerontol Psychol Sci Soc Sci.

[34] Phillips S, McAuley E (2012). Social cognitive influences on physical activity participation in long-term breast cancer survivors. Psychooncology.

[35] Rovniak LS, Anderson ES, Winett RA, Stephens RS (2002). Social cognitive determinants of physical activity in young adults: a prospective structural equation analysis. Ann Behav Med.

[36] Ayotte B, Margrett J, Hicks-Patrick J (2010). Physical activity in middle-aged and young-old adults: the roles of self-efficacy, barriers, outcome expectancies, self-regulatory behaviors and social support. J Health Psychol.

[37] Anderson-Bill ES, Winett RA, Wojcik JR (2011). Social cognitive determinants of nutrition and physical activity among web-health users enrolling in an online intervention: the influence of social support, self-efficacy, outcome expectations, and self-regulation. J Med Internet Res.

[38] Anderson E, Winett RA, Wojcik JR (2007). Self-regulation, self-efficacy, outcome expectations, and social support: social cognitive theory and nutrition behavior. Ann Behav Med.

[39] Graves KD (2003). Social cognitive theory and cancer patients’ quality of life: a meta-analysis of psychosocial intervention components. Health Psychol.

[40] Courneya KS, Segal RJ, Mackey JR, Gelmon K, Reid RD, Friedenreich CM (2007). Effects of aerobic and resistance exercise in breast cancer patients receiving adjuvant chemotherapy: a multicenter randomized controlled trial. J Clin Oncol.

[41] Courneya KS, Sellar CM, Stevinson C, McNeely ML, Peddle CJ, Friedenreich CM (2009). Randomized controlled trial of the effects of aerobic exercise on physical functioning and quality of life in lymphoma patients. J Clin Oncol.

[42] Rowland JH, Hewitt M, Ganz PA (2006). Cancer survivorship: a new challenge in delivering quality cancer care. J Clin Oncol.

[43] Courneya KS (2010). Efficacy, effectiveness, and behavior change trials in exercise research. Int J Behav Nutr Phys.

[44] Liberati A, Altman DG, Tetzlaff J, Mulrow C, Gotzsche PC, Ioannidis JPA, et al. The PRISMA statement for reporting systematic reviews and meta-analyses of studies that evaluate healthcare interventions: explanation and elaboration. BMJ. 2009;339. 10.1136/bmj.b2700 PMC2714672 19622552

[45] Review Manager (RevMan). The Cochrane Collaboration, Copenhagen, Denmark. 2011.

[46] Deeks JJ, Higgins JPT, Altman DG, Higgins JPT, Green S (2008). Analysing data and undertaking meta-analyses. Cochrane handbook for systematic reviews of interventions.

[47] Thomas BH, Ciliska D, Dobbins M, Micucci S (2004). A process for systematically reviewing the literature: providing the research evidence for public health nursing interventions. Worldviews Evid Based Nurs.

[48] Hatchett A, Hallam J, Ford M (2012). Evaluation of a social cognitive theory-based email intervention designed to influence the physical activity of survivors of breast cancer. Psychooncology.

[49] Ligibel JA, Meyerhardt J, Pierce JP, Najita J, Shockro L, Campbell N (2011). Impact of a telephone-based physical activity intervention upon exercise behaviors and fitness in cancer survivors enrolled in a cooperative group setting. Breast Cancer Res Treat.

[50] Pinto BM (2005). Home-based physical activity intervention for breast cancer patients. J Clin Oncol.

[51] Rogers L, Hopkins-Price P, Vicari S, Pamenter R, Courneya K, Markwell S (2009). A randomized trial to increase physical activity in breast cancer survivors. Med Sci Sports Exerc.

[52] Wang Y-J, Boehmke M, Wu Y-WB, Dickerson SS, Fisher N (2011). Effects of a 6-week walking program on Taiwanese women newly diagnosed with early-stage breast cancer. Cancer Nurs.

[53] Pinto BM, Papandonatos GD, Goldstein MG, Marcus BH, Farrell N (2013). Home-based physical activity intervention for colorectal cancer survivors. Psychooncology.

[54] Short CE, James EL, Girgis A, D’Souza MI, Plotnikoff RC. Main outcomes of the Move More for Life trial: a randomised controlled trial examining the effects of tailored-print and targeted-print materials for promoting physical activity among post-treatment breast cancer survivors. Psychooncology. 2014. 10.1002/pon.3639 25060288

[55] Short CE, James EL, Girgis A, Mcelduff P, Plotnikoff RC (2012). Move more for life: the protocol for a randomised efficacy trial of a tailored-print physical activity intervention for post-treatment breast cancer survivors. BMC Cancer.

[56] Valle CG, Tate DF, Mayer DK, Allicock M, Cai J (2013). A randomized trial of a Facebook-based physical activity intervention for young adult cancer survivors. J Cancer Surviv.

[57] Bennett JA, Lyons KS, Winters-Stone K, Nail LM, Scherer J (2007). Motivational interviewing to increase physical activity in long-term cancer survivors: a randomized controlled trial. Nurs Res.

[58] Matthews CE, Wilcox S, Hanby CL, Der Ananian C, Heiney SP, Gebretsadik T (2007). Evaluation of a 12-week home-based walking intervention for breast cancer survivors. Support Care Cancer.

[59] Parsons JK, Newman VA, Mohler JL, Pierce JP, Flatt S, Marshall J (2008). Dietary modification in patients with prostate cancer on active surveillance: a randomized, multicentre feasibility study. BJU Int.

[60] Demark-Wahnefried W, Case LD, Blackwell K, Marcom PK, Kraus W, Aziz N (2008). Results of a diet/exercise feasibility trial to prevent adverse body composition change in breast cancer patients on adjuvant chemotherapy. Clin Breast Cancer.

[61] Campbell MK, Carr C, Devellis B, Switzer B, Biddle A, Amamoo MA (2009). A randomized trial of tailoring and motivational interviewing to promote fruit and vegetable consumption for cancer prevention and control. Ann Behav Med.

[62] Demark-Wahnefried W, Clipp E, McBride C, Lobach D, Lipkus I, Peterson B (2003). Design of FRESH START: a randomized trial of exercise and diet among cancer survivors. Med Sci Sports Exerc.

[63] Demark-Wahnefried W, Clipp EC, Lipkus IM, Lobach D, Snyder DC, Sloane R (2007). Main outcomes of the FRESH START trial: a sequentially tailored, diet and exercise mailed print intervention among breast and prostate cancer survivors. J Clin Oncol.

[64] Djuric Z, Ellsworth JS, Weldon AL, Ren J, Richardson CR, Resnicow K (2011). A diet and exercise intervention during chemotherapy for breast cancer. Open Obes J.

[65] von Gruenigen VE, Courneya KS, Gibbons HE, Kavanagh MB, Waggoner SE, Lerner E (2008). Feasibility and effectiveness of a lifestyle intervention program in obese endometrial cancer patients: a randomized trial. Gynecol Oncol.

[66] Djuric Z, DiLaura NM, Jenkins I, Darga L, Jen CK-L, Mood D (2002). Combining weight-loss counseling with the weight watchers plan for obese breast cancer survivors. Obes Res.

[67] von Gruenigen V, Frasure H, Kavanagh MB, Janata J, Waggoner S, Rose P (2012). Survivors of uterine cancer empowered by exercise and healthy diet (SUCCEED): a randomized controlled trial. Gynecol Oncol.

[68] Rogers L, Hopkins Price P, Vicari S, Markwell S, Pamenter R, Courneya K (2009). Physical activity and health outcomes three months after completing a physical activity behavior change intervention: persistent and delayed effects. Cancer Epidemiol Biomarkers Prev.

[69] Rogers L, Vicari S, Courneya K (2010). Lessons learned in the trenches: facilitating exercise adherence among breast cancer survivors in a group setting. Cancer Nurs.

[70] Rogers LQ, Markwell S, Hopkins-Price P, Vicari S, Courneya K, Hoelzer K (2011). Reduced barriers mediated physical activity maintenance among breast cancer survivors. J Sport Exerc Psychol.

[71] Pinto BM, Rabin C, Dunsiger S (2009). Home-based exercise among cancer survivors: adherence and its predictors. Psychooncology.

[72] Rabin CS, Pinto BM, Trunzo JJ, Frierson GM, Bucknam LM (2006). Physical activity among breast cancer survivors: regular exercisers vs participants in a physical activity intervention. Psychooncology.

[73] von Gruenigen V, Gibbons H, Kavanagh M, Janata J, Lerner E, Courneya K (2009). A randomized trial of a lifestyle intervention in obese endometrial cancer survivors: quality of life outcomes and mediators of behavior change. Health Qual Life Outcomes.

[74] Christy SM, Mosher CE, Sloane R, Snyder DC, Lobach DF, Demark-Wahnefried W (2011). Long-term dietary outcomes of the FRESH START intervention for breast and prostate cancer survivors. J Am Diet Assoc.

[75] Michie S, Ashford S, Sniehotta FF, Dombrowski SU, Bishop A, French DP (2011). A refined taxonomy of behaviour change techniques to help people change their physical activity and healthy eating behaviours: the CALO-RE taxonomy. Psychol Health.

[76] Michie S, Richardson M, Johnston M, Abraham C, Francis J, Hardeman W (2013). The behavior change technique taxonomy (v1) of 93 hierarchically clustered techniques: building an international consensus for the reporting of behavior change interventions. Ann Behav Med.

[77] Norris JM, Culos-Reed SN, Carlson LE, Aldous SH (2007). Utilizing the TPB for understanding yoga participation in cancer survivors. J Sport Exerc Psychol.

[78] Mosher CE, Fuemmeler BF, Sloane R, Kraus WE, Lobach DF, Snyder DC (2008). Change in self-efficacy partially mediates the effects of the FRESH START intervention on cancer survivors’ dietary outcomes. Psychooncology.

[79] Cohen J (1988). Statistical power analysis for the behavioral sciences.

[80] Greaves CJ, Sheppard KE, Abraham C, Hardeman W, Roden M, Evans PH (2011). Systematic review of reviews of intervention components associated with increased effectiveness in dietary and physical activity interventions. BMC Public Health.

[81] Michie S, Abraham C, Whittingham C, McAteer J, Gupta S (2009). Effective techniques in healthy eating and physical activity interventions: a meta-regression. Health Psychol.

[82] Short CE, James EL, Stacey F, Plotnikoff RC. A qualitative synthesis of trials promoting physical activity behaviour change among post-treatment breast cancer survivors. J Cancer Surviv. 2013. 10.1007/s11764-013-0296-4 PMC3838584 23888337

[83] Spark LC, Reeves MM, Fjeldsoe BS, Eakin EG. Physical activity and/or dietary interventions in breast cancer survivors: a systematic review of the maintenance of outcomes. J Cancer Surviv. 2013. 10.1007/s11764-012-0246-6 23179496

[84] Nigg CR, Long CR (2012). A systematic review of single health behavior change interventions vs. multiple health behavior change interventions among older adults. TBM.

[85] Webb TL, Sheeran P (2006). Does changing behavioral intentions engender behavior change? A meta-analysis of the experimental evidence. Psychol Bull.

[86] Williams DM, Anderson ES, Winett RA (2005). A review of the outcome expectancy construct in physical activity research. Ann Behav Med.

[87] Anderson ES, Winett RA, Wojcik JR, Williams DM (2010). Social cognitive mediators of change in a group randomized nutrition and physical activity intervention: social support, self-efficacy, outcome expectations and self-regulation in the guide-to-health trial. J Health Psychol.

[88] Prestwich A, Sniehotta FF, Whittington C, Dombrowski SU, Rogers L, Michie S (2013). Does theory influence the effectiveness of health behavior interventions? Meta-analysis. Health Psychol.

[89] Avery KNL, Donovan JL, Horwood J, Lane JA (2013). Behavior theory for dietary interventions for cancer prevention: a systematic review of utilization and effectiveness in creating behavior change. Cancer Causes Control.

[90] Broekhuizen K, Kroeze W, Poppel MNM, Oenema A, Brug J (2012). A systematic review of randomized controlled trials on the effectiveness of computer-tailored physical activity and dietary behavior promotion programs: an update. Ann Behav Med.

[91] Moher D, Hopewell S, Schulz KF, Montori V, Gotzsche PC, Devereaux PJ (2010). CONSORT 2010 explanation and elaboration: updated guidelines for reporting parallel group randomised trials. BMJ.

[92] Higgins JPT, Green S. Cochrane handbook for systematic reviews of interventions version 5.1.0 [updated March 2011]: The Cochrane Collaboration; 2011. Available from: www.cochrane-handbook.org.

[93] Livingston PM, Salmon J, Courneya KS, Gaskin CJ, Craike M, Botti M (2011). Efficacy of a referral and physical activity program for survivors of prostate cancer [ENGAGE]: rationale and design for a cluster randomised controlled trial. BMC Cancer.

[94] Rogers LQ, McAuley E, Anton PM, Courneya KS, Vicari S, Hopkins-Price P (2012). Better exercise adherence after treatment for cancer (BEAT Cancer) study: rationale, design, and methods. Contemp Clin Trials.

[95] James EL, Stacey F, Chapman K, Lubans DR, Asprey G, Sundquist K (2011). Exercise and nutrition routine improving cancer health (ENRICH): the protocol for a randomized efficacy trial of a nutrition and physical activity program for adult cancer survivors and carers. BMC Public Health.

[96] Blackburn GL, Wang KA (2007). Dietary fat reduction and breast cancer outcome: results from the Women’s Intervention Nutrition Study (WINS). Am J Clin Nutr.

[97] Chlebowski RT, Blackburn GL, Thomson CA, Nixon DW, Shapiro A, Hoy MK (2006). Dietary fat reduction and breast cancer outcome: interim efficacy results from the Women’s Intervention Nutrition Study. J Natl Cancer Inst.

[98] Hoy MK, Winters BL, Chlebowski RT, Papoutsakis C, Shapiro A, Lubin MP (2009). Implementing a low-fat eating plan in the Women’s Intervention Nutrition Study. J Am Diet Assoc.

[99] Pierce JP, Natarajan L, Caan BJ, Parker BA, Greenberg RE, Flatt SW (2007). Influence of a diet very high in vegetables, fruit, and fiber and low in fat on prognosis following treatment for breast cancer: The Women’s Healthy Eating and Living (WHEL) randomized trial. J Am Med Assoc.

[100] Pierce JP, Natarajan L, Sun S, Al-Delaimy W, Flatt SW, Kealey S (2006). Increases in plasma carotenoid concentrations in response to a major dietary change in the Women’s Healthy Eating and Living study. Cancer Epidemiol Biomarkers Prev.

[101] Carmack Taylor CL, Demoor C, Smith MA, Dunn AL, Basen-Engquist K, Nielsen I (2006). Active for Life After Cancer: a randomized trial examining a lifestyle physical activity program for prostate cancer patients. Psychooncology.

[102] Morey MC, Snyder DC, Sloane R, Cohen HJ, Peterson B, Hartman TJ (2009). Effects of home-based diet and exercise on functional outcomes among older, overweight long-term cancer survivors: RENEW: a randomized controlled trial. J Am Med Assoc.

[103] Snyder DC, Morey MC, Sloane R, Stull V, Cohen HJ, Peterson B (2009). Reach out to Enhance Wellness in Older Cancer Survivors (RENEW): design, methods and recruitment challenges of a home-based exercise and diet intervention to improve physical function among long-term survivors of breast, prostate, and colorectal cancer. Psychooncology.

[104] Twiss JJ, Waltman NL, Berg K, Ott CD, Gross GJ, Lindsey AM (2009). An exercise intervention for breast cancer survivors with bone loss. J Nurs Scholarsh.

[105] Michie S, Prestwich A (2010). Are interventions theory-based? Development of a theory coding scheme. Health Psychol.

[106] Strasser B, Steindorf K, Wiskemann J, Ulrich CM (2013). Impact of resistance training in cancer survivors: a meta-analysis. Med Sci Sports Exerc.

[107] Hutchison AJ, Breckon JD, Johnston LH (2008). Physical activity behavior change interventions based on the transtheoretical model: a systematic review. Health Educ Behav.

[108] Painter JE, Borba CPC, Hynes M, Mays D, Glanz K (2008). The use of theory in health behavior research from 2000 to 2005: a systematic review. Ann Behav Med.

[109] Lawler SP, Winkler E, Reeves MM, Owen N, Graves N, Eakin EG (2010). Multiple health behavior changes and co-variation in a telephone counseling trial. Ann Behav Med.

[110] White SM, McAuley E, Estabrooks PA, Courneya KS (2009). Translating physical activity interventions for breast cancer survivors into practice: an evaluation of randomized controlled trials. Ann Behav Med.

[111] Reedy J, Haines PS, Campbell MK (2005). The influence of health behavior clusters on dietary change. Prev Med.

[112] Ko LK, Campbell MK, Lewis MA, Earp J, DeVellis B (2010). Mediators of fruit and vegetable consumption among colorectal cancer survivors. J Cancer Surviv.

[113] Wilkinson AV, Barrera SL, McBride CM, Snyder DC, Sloane R, Meneses KM (2012). Extant health behaviors and uptake of standardized vs tailored health messages among cancer survivors enrolled in the FRESH START trial: a comparison of fighting-spirits vs fatalists. Psychooncology.

